# Supplementary material for: Uptake and use of a minimum data set (MDS) for older people living and dying in care homes in England: a realist review protocol
Source: BMJ Open. 2020 Nov 14;10(11):e040397. doi: 10.1136/bmjopen-2020-040397 (PMC7668360; doi:10.1136/bmjopen-2020-040397)
Supplement: Supplementary data [file bmjopen-2020-040397supp001.pdf]

Supplementary Table S1: Proposed search terms for care homes minimum data set (MDS)

1. Combined searches for scoping review and process evaluation review (DACHA Study's workpackage-1)

1. *Homes for the Aged/
2. *Nursing Homes/
3. *Long-Term Care/
4. *Residential Facilities/
5. *Respite Care/
6. *Intermediate Care/
7. "care home$".ab,ti.
8. "nursing home$".ab,ti.
9. "residential care".ab,ti.
10. ("long term care" or "long-term care" or "longterm care").ab,ti.
11. "home$ for the aged".ab,ti.
12. "care facilit*".ab,ti.
13. "old$ people$ home$".ti,ab.
14. (retir$ adj2 home$).ab,ti.
15. ("old$ adult$" adj3 (facilit$ or residential or accommodation)).ab,ti.
16. ("old$ people$" adj3 (facilit$ or residential or accommodation)).ab,ti.
17. ("old$ person$" adj3 (facilit$ or residential or accommodation)).ab,ti.
18. ((geriatric$ or elder$ or senior$ or retir$) adj3 (facilit$ or residential or accommodation)).ab,ti.
19. "respite care".ti,ab.
20. "intermediate care".ti,ab.
21. or/1-20
22. *Randomized Controlled Trials as Topic/
23. Randomized controlled trial/
24. Random allocation/
25. Double blind method/
26. Single blind method/
27. Clinical Trial/
28. Clinical trials as Topic/
29. "randomi*ed".ab,ti.
30. randomly.ab,ti.
31. controlled clinical trial.pt.
32. Evaluation Study/
33. Comparative Study/
34. "before and after study".ti,ab,mp.
35. or/22-34
36. "Outcome and Process Assessment (Health Care)"/
37. *Implementation Science/
38. "process evaluation".ab,ti.
39. (process$ adj3 evaluation$).ab,ti.
40. (program$ adj3 evaluation$).ab,ti.
41. implementation.ab,ti.
42. context$.ab,ti.
43. fidelity.ab,ti.
44. or/36-43
45. *Qualitative Research/
46. *Focus Groups/
47. *Interviews as Topic/
48. *Narration/
49. (("semi-structured" or semistructured or unstructured or informal or "in-depth" or "indepth" or "face to face" or structured or guide) adj3 (interview$ or discussion$ or questionnaire$)).ab,ti.
50. or/45-49
51. 44 or 50
52. 21 and 35
53. 51 and 52
54. limit 52 to yr="2009 -Current"
55. 53 or 54

2. Hits from the above searches will be downloaded into Endnote; we will then search in Endnote for MDS related studies using the following terms:

MDS
Minimum Data Set
Minimum Data
Inter-RAI
RAI
Research Assessment Instrument
