# Supplementary material for: Uptake and use of a minimum data set (MDS) for older people living and dying in care homes in England: a realist review protocol
Source: BMJ Open. 2020 Nov 14;10(11):e040397. doi: 10.1136/bmjopen-2020-040397 (PMC7668360; doi:10.1136/bmjopen-2020-040397)
Supplement: Author's manuscript [file bmjopen-2020-040397.draft_revisions.pdf]

BMJ Open is committed to open peer review. As part of this commitment we make the peer review history of every article we publish publicly available.

When an article is published we post the peer reviewers' comments and the authors' responses online. We also post the versions of the paper that were used during peer review. These are the versions that the peer review comments apply to.

The versions of the paper that follow are the versions that were submitted during the peer review process. They are not the versions of record or the final published versions. They should not be cited or distributed as the published version of this manuscript.

BMJ Open is an open access journal and the full, final, typeset and author-corrected version of record of the manuscript is available on our site with no access controls, subscription charges or pay-per-view fees (http://bmjopen.bmj.com).

If you have any questions on BMJ Open's open peer review process please email info.bmjopen@bmj.com

BMJ Open

The uptake and use of a minimum data set (MDS) for older people living and dying in care homes in England – a realist review protocol

Journal:	*BMJ Open*
Manuscript ID	bmjopen-2020-040397
Article Type:	Protocol
Date Submitted by the Author:	14-May-2020

Complete List of Authors:	Musa, Massirfufulay; University of Hertfordshire, Centre for Research in Public Health and Community Care (CRIPACC); King's College London, Faculty of Nursing, Midwifery and Palliative Care<br>Akdur, Gizdem ; University of Hertfordshire, Centre for Research in Public health and Community Care (CRIPACC)<br>Hanratty, Barbara; Newcastle University, Institute of Health and Society / Newcastle University Institute for Ageing<br>Kelly, Sarah; University of Cambridge, Institute of Public Health<br>Gordon, Adam; University of Nottingham, Division of Rehabilitation and Ageing<br>Peryer, Guy; University of East Anglia, Faculty of Medicine and Health Sciences<br>Spilsbury, Karen; University of Leeds, School of Healthcare<br>Killett, Anne; University of East Anglia, Faculty of Medicine and Health Sciences<br>Burton, Jennifer ; University of Glasgow, Institute of Cardiovascular and Medical Sciences<br>Meyer, Julienne; City University of London, National Care Forum<br>Fortescue, Sue; Alzheimer Society Research Network<br>Towers, Ann-Marie; University of Kent, Centre for Health Services Studies<br>Irvine, Lisa; University of Hertfordshire, Centre for Research in Public health and Community Care (CRIPACC)<br>Goodman, Claire; University of Hertfordshire, Centre for Research in Public health and Community Care (CRIPACC)

Keywords:	HEALTH SERVICES ADMINISTRATION & MANAGEMENT, Quality in health care < HEALTH SERVICES ADMINISTRATION & MANAGEMENT, PALLIATIVE CARE, PUBLIC HEALTH, EPIDEMIOLOGY, GERIATRIC MEDICINE

SCHOLARONE™
Manuscripts

I, the Submitting Author has the right to grant and does grant on behalf of all authors of the Work (as defined in the below author licence), an exclusive licence and/or a non-exclusive licence for contributions from authors who are: i) UK Crown employees; ii) where BMJ has agreed a CC-BY licence shall apply, and/or iii) in accordance with the terms applicable for US Federal Government officers or employees acting as part of their official duties; on a worldwide, perpetual, irrevocable, royalty-free basis to BMJ Publishing Group Ltd ("BMJ") its licensees and where the relevant Journal is co-owned by BMJ to the co-owners of the Journal, to publish the Work in this journal and any other BMJ products and to exploit all rights, as set out in our licence.

The Submitting Author accepts and understands that any supply made under these terms is made by BMJ to the Submitting Author unless you are acting as an employee on behalf of your employer or a postgraduate student of an affiliated institution which is paying any applicable article publishing charge ("APC") for Open Access articles. Where the Submitting Author wishes to make the Work available on an Open Access basis (and intends to pay the relevant APC), the terms of reuse of such Open Access shall be governed by a Creative Commons licence – details of these licences and which Creative Commons licence will apply to this Work are set out in our licence referred to above.

Other than as permitted in any relevant BMJ Author's Self Archiving Policies, I confirm this Work has not been accepted for publication elsewhere, is not being considered for publication elsewhere and does not duplicate material already published. I confirm all authors consent to publication of this Work and authorise the granting of this licence.

For peer review only

Title page

Title:

The uptake and use of a minimum data set (MDS) for older people living and dying in care homes in England – a realist review protocol

Corresponding Author: Claire Goodman

Centre for Research in Public health and Community Care (CRIPACC) University of Hertfordshire AL10 9AB

E-mail: c.goodman@herts.ac.uk

Key words: Care Home, minimum data set, geriatric assessment, nursing tools, older people care

Word count: Abstract (302); main text (2630), references (1283)

Contributing authors: Please insert your contact details

Authors	E-mail addresses	Address
Mr Massirfufulay Kpehe Musa	m.musa2@herts.ac.uk	Centre for Research in Public health and Community Care (CRIPACC) University of Hertfordshire AL10 9AB
Dr Gizdem Akdur	g.akdur@herts.ac.uk	Centre for Research in Public health and Community Care (CRIPACC) University of Hertfordshire AL10 9AB
Professor Barbara Hanratty	barbara.hanratty@newcastle.ac.uk	Population Health Sciences Institute Biomedical Research Building (Second floor) Campus for Ageing and Vitality Newcastle University Newcastle upon Tyne NE4 5PL
Dr Sarah Kelly	sarah.kelly@thisinstitute.cam.ac.uk	Institute of Public Health University of Cambridge CB2 0SR
Dr Adam Gordon	adam.gordon@nottingham.ac.uk	School of Medicine University of Nottingham DE22 3DT
Dr Guy Peryer	g.peryer@uea.ac.uk	Faculty of Medicine and Health Sciences,

For peer review only - http://bmjopen.bmj.com/site/about/guidelines.xhtml

		University of East Anglia, NR4 7TJ
Professor Karen Spilsbury	k.spilsbury@leeds.ac.uk	School of Healthcare, Faculty of Medicine and Health, University of Leeds, Leeds, LS2 9JT
Dr Anne Killett	a.killett@uea.ac.uk	Faculty of Medicine and Health Sciences University of East Anglia Norwich Research Park NR4 7TJ
Dr Jennifer Burton	Jenni.Burton@glasgow.ac.uk	Institute of Cardiovascular and Medical Sciences, University of Glasgow, New Lister Building, Glasgow Royal Infirmary, G31 2ER
Professor Julienne Meyer	J.Meyer@city.ac.uk	National Care Forum
Ms Sue Fortescue	sfortescue@hotmail.co.uk	Alzheimer Society Research Network
Ms Ann-Marie Towers	a.towers@kent.ac.uk	Centre for Health Services Studies University of Kent CT2 7NF
Ms Lisa Irvine	l.irvine@herts.ac.uk	Centre for Research in Public health and Community Care (CRIPACC) University of Hertfordshire AL10 9AB
Professor Claire Goodman	c.goodman@herts.ac.uk	Centre for Research in Public health and Community Care (CRIPACC) University of Hertfordshire AL10 9AB

For peer review only

ABSTRACT

Introduction: Care homes provide nursing and social care for older people who can no longer live independently at home. In the UK, there is no consistent approach to how information about residents' medical history, care needs, and preferences are collected and shared. This limits opportunities to understand the care home population, have a systematic approach to assessment and documentation of care, identify care home residents at risk of deterioration, and review care. In many countries, resident Minimum Data Sets (MDS) provide a standardised way to address this issue and help to improve residents' outcomes and inform care commissioning. Less well documented is the process of implementation and how different groups: residents, family, staff, commissioners, regulators, health and social care organisations interpret and use an MDS generated information.

Methods and analysis: A realist review to develop a conceptual understanding of what supports the implementation and use of an MDS in care homes. Stage one will scope the literature and consult with relevant stakeholders to develop explanatory theories of what ensures effective uptake and sustained implementation of an MDS. Stage two will test and refine these theories through further iterative searches of the evidence to establish how effective uptake of an MDS can be achieved. Stage three will synthesise the evidence to develop a programme theory of how an MDS works for different stakeholders and in what circumstances.

Ethics and dissemination: The protocol has been submitted to the University of Hertfordshire Ethics Committee. Findings will be disseminated through briefings with stakeholders, conference presentations, a national consultation on the use of an MDS in UK long-term care settings, and publications in peer-reviewed journals.

Review registration number: It is registered on the International Prospective Register of Systematic Reviews (PROSPERO) - CRD42020171323 (registered 09/03/2020).

Keywords: Care Home, minimum data set, geriatric assessment, nursing tools, older people care

For peer review only

Box 1: Strengths and limitations of this study

- The review will identify how, why and in what circumstances the implementation of an MDS in long term care settings leads to resident, staff and service benefits.
- The synthesis will integrate qualitative and quantitative evidence that offers transferable learning for long term care settings that do not currently use an MDS.
- The synthesis will provide guidance to improve implementation of an MDS that accounts for the heterogeneity of the long term care sector
- The programme theories will reflect how care homes use and share residents' data, the role of the regulator and legal frameworks and what is required for data sharing within and across organisations.
- There are time constraints that may result in the team focusing on or prioritising some aspects of an MDS implementation over others.

For peer review only

BACKGROUND

There are nearly 12 million (11,989,322) people aged 65 years and above in the United Kingdom (UK) of which an estimated 1.6 million are aged 85 years and above and more than 500,000 (579,776) people are aged 90 years and above.[1] However, with greater longevity (e.g. age 85 years and above) comes higher levels of dependency, dementia and comorbidity,[2] which in turn intensify the need for social care services.[3] Approximately 420,000 older people in England and Wales live in care homes. [4] The care home population represents the oldest and most vulnerable group of older people, with approximately 70% of them living with cognitive impairment, [5 6] and 76% requiring assistance with mobility.[7]

In the UK, there is no consistent approach to how information about residents' medical history, care needs, and preferences are collected and used. The absence of a national mandate, lack of links with National Health Services (NHS) data, and implementation challenges have meant that the minimum data set (MDS) and data-driven approaches to resident assessment have been limited to single projects.[8] The lack of a link between care home data and the NHS data recently became evident when figures reported by the Office for National Statistics during the first three weeks of Covid-19 underestimated impact of the pandemic among care home residents.[9] All care homes, however, routinely collect large amounts of data about their residents. The challenge is to establish systems of assessment and recording that are evidence-based, accessible and valuable to those using, providing, commissioning and regulating care services. Without a unified record there are duplication of assessments, communication failures and, unmet care needs.[10 11] Over the next two decades older

For peer review only - http://bmjopen.bmj.com/site/about/guidelines.xhtml

people likely to need long term care will increase;[2] determining consistent ways to assess and document care for residents in these settings are a priority.

The use of minimum data sets (MDS) have been adopted in several countries and in many countries the MDS are mandated and/or linked to national reimbursement systems and quality monitoring. One international consortium of researchers and practitioners have developed instruments that share common language (clinical concepts) about patients/residents for use in different health and social care settings (International Resident Assessment Instrument (InterRAI)),[12] including one developed for long term care facilities (LTCF).[13] Research has demonstrated the value of minimum data sets (MDS) like Inter-RAI or its equivalent to commissioners and service providers in enabling identification of care needs and residents at risk of ill health.[14-18] They provide a comprehensive account of resident characteristics, resource use, and care outcomes in key areas (e.g. activities of daily living, cognitive performance, pain, cost of care, and infection).[19] What is less clear is how their use affects the everyday work and care practices of care home staff and how the different priorities of social care providers, residents and family members, clinicians and commissioner are met. Nor is it known what resources are required to sustain the system within a care home, and how staff or residents themselves act on resident data for the delivery of personalised care. [13 14 19-21]

A minimum data set (MDS) in this realist review is defined as a comprehensive, standardised account of the characteristics and needs of residents living in long-term care settings. The review recognises the benefits of standardised approaches to capturing residents' data and concentrates on how an MDS is used by staff and what supports effective uptake among staff members. It also takes account of the involvement of residents themselves and their family in influencing how an MDS is used.

For peer review only - http://bmjopen.bmj.com/site/about/guidelines.xhtml

METHODS AND ANALYSIS

Review objective

To develop a theory-driven understanding of how internationally deployed Minimum Data Sets (MDS) are implemented in care home settings and how their use by care home staff influences outcomes for residents, family, staff and linked organisations.

To offer transferrable learning and/or utility for an MDS development in countries that do not have an MDS for older people in long term care settings.

Review questions

1. What are the key features or elements of an MDS that influence implementation and ongoing use of an MDS in care homes?
2. How do care home staff use an MDS to inform care of their residents?
3. What outcomes (including resource use) does the use of an MDS produce for care home staff, residents and their families?
4. What would need to be in place for an MDS implementation?

Study design

A realist review is an interpretive, theory-driven evidence review.[22 23] Interventions like the minimum data set (MDS) will always rely on human agency to affect change. A realist approach argues that the features or elements of the intervention will produce a range of potential responses to the intervention which will impact on the outcomes. It assumes that there is a knowable, independent reality that will shape how different participants react to an intervention, whether they are aware of these influences or not.[24] Thus, uptake and implementation of an MDS can lead to different outcomes for different stakeholders (e.g. residents, staff, commissioners, regulator) depending on who is involved, the resources available and how the MDS is used.[25 26]

Using a realist approach, there are the four key linked concepts for explaining and building a theory of how a programme works: (i) contexts (C), which are often the 'backdrop' of interventions;[22] (ii) mechanisms (M), which are not observed directly but account for what it is about programmes that make them work,[27 28] characterised as *"a process that bring about or prevents some change in a concrete system"*[29] (iii) outcomes (O) of the intervention (planned or unplanned, visible or not) or

For peer review only - http://bmjopen.bmj.com/site/about/guidelines.xhtml

strategies of the intervention;[30] It is the Context-mechanism-outcome (CMO) configurations (models indicating how programmes activate mechanisms for whom and in what conditions, to elicit outcomes) that are the building blocks of the theory.[23] Thus, in care home settings, staff understanding of their responsibility for completing an MDS could be a context (C), which triggers how staff prioritise recording information as part of care work (M) to identify residents at risk of deterioration (O).

This review will follow the Realist And Meta-narrative Evidence Syntheses: Evolving Standards (RAMESES).[31] Organised in three stages, we will first undertake a scoping of the literature to identify care home specific work on the uptake of MDS and develop relevant theories around staff uptake and implementation and outcomes specific to the use of an MDS in long term (care home) settings. Stage two will test and refine the emergent theories that underpin the use of an MDS and that leads to both intended and unintended outcomes for staff and residents. Stage three will synthesise the findings to establish how and when the use of an MDS achieves different outcomes for residents, families, staff and organisations.

Stage 1: Defining the scope of the review, identifying existing theories and theory development

This review is nested within a larger review ("*A systematic review of process and contextual factors that influence research implementation in care homes and identification of key measures and outcomes in care home research*"; PROSPERO reference: CRD42020155923). The literature identified from the larger review is the basis for the scoping work and identification of studies that have used MDS, or similar approaches to document resident, staff and organisational outcomes. We will develop a series of 'if-then statements' to summarise the dominant arguments and supporting evidence of what supports the uptake and use of MDS in long-term care settings. This will inform the development of hypotheses that posit possible contexts (C) that are the backdrop to successful (or not) uptake[22] of MDS; the mechanisms (M) they trigger[29] and planned or unplanned Outcomes (O) arising from the use of MDS.[30] Possible context-mechanism-outcome (CMO) configurations will be discussed across the research team and subject experts on MDS and inform the stage two of the review phase and additional searches.

There are several ways to conceptualise the development and uptake of MDS as many have their roots in medical approaches to assessment and health systems design. It is therefore likely that the review

For peer review only - http://bmjopen.bmj.com/site/about/guidelines.xhtml

will be informed by and aim to build on theories of implementation in long-term care,[29 32] uptake of technological innovation[33 34] assessment of older people with complex needs,[2 4 7] person-centred care,[35 36] and risk management and quality assurance.[37]

The introduction of an MDS in care homes is sensitive to the resource and policy constraints under which the care homes operate. Candidate theories will therefore consider the role of the regulator and legal frameworks that incentivise (or not) data sharing across organisations.

The scoping of the literature will focus on studies that report on how an MDS is used in long term care settings. Outcomes of interest will be established by the project team as an iterative process but are likely to include evidence of its impact on: accuracy of reporting, needs assessment, staff workload, quality of care, resource use, staff satisfaction and access to care. No geographical restrictions will apply, although we will only include studies that are published in English and focus on the uptake and use of an MDS in long term care settings.

Literature selection criteria and quality appraisal

There will be no restriction on the types of study design for eligibility.[38] Article selection will be based on the extent to which research on the uptake and routine use of an MDS can contribute to the development of a programme theory of implementation of MDS in long term care settings.

Included studies are likely to cover the following:

- Studies on the introduction and development of an MDS with care home staff
- Studies that focus on the inclusion and engagement of care home staff, residents and their representatives in sharing resident data with the specific remit of improving resident outcomes.
- Implementation studies that provide evidence on what facilitates and inhibits the shared documentation and care planning in care home setting including digital innovation.
- Studies on commissioning services for care homes based on care home generated data

For peer review only - http://bmjopen.bmj.com/site/about/guidelines.xhtml

Methodological quality of included papers will be carried out in accordance with previous appraisal work within a realist project.[26] Papers will be appraised based on their relevance to the review questions and rigour. Two team members (MM GA), in consultation with CG and the wider group will lead this process. Weaker papers and those with equivocal or negative findings will be considered if they contribute to the overall programme theories.

Data synthesis

Data synthesis of the scoping phase will use realist logic of analysis to align data from each eligible article with possible context-mechanism-outcome (CMO) configurations, or specific elements that answer the research questions. These emerging findings, and putative patterns of association within the data will be tested further in stage two, to build causal explanations based on the observed interactions between context, mechanism, and outcomes.

Stage 2: Testing and refining the programme theories

Further iterative searches of the evidence will be directly informed by the CMOs developed in stage one as candidate programme theories. The iterative circle will continue throughout the course of the review until theoretical saturation has been achieved. [31 39]

Data will be extracted using a bespoke data extraction form. It will include descriptive data on study characteristics and is likely to focus on what can be learnt about the role and work of staff, the resources required to implement an MDS, the features of the settings (e.g., workforce capacity, size of care homes), explicit and implicit theories for how interventions were anticipated to work, and patient and carer outcomes. A sample of the papers, including those that appear to offer most learning and their completed data extraction forms will be shared across the project team to support ongoing discussion and debate of the candidate theory(ies) and their supporting evidence.

Stage 3: Analysis and synthesis of evidence from the proposed programme theories

To support hypothesis refinement and "fine tune" the theory(ies) that show the most promise, we will further test findings from stage two in a series of interviews.[40] We will do this through stakeholders' consultation (Box 2). Four group interviews with frontline staff will take place including care home staff, managers, including staff from care homes who use predominately paper based records and staff who routinely use electronic records for their residents. We will also carry out individual semi-

For peer review only - http://bmjopen.bmj.com/site/about/guidelines.xhtml

structured interviews with up to eight stakeholders who are experts regarding the use of care home residents' data.

To reject or further refine the proposed programme theories, we will sense check by structuring the proposed programme theories with the supporting evidence for discussion with the whole research project team.

Box 2: Stakeholders' consultation

During stakeholders' consultation interviews, we will explore:

i) The fit between the emerging programme theories and how stakeholders understand what is needed for the development and use of MDS in long term care settings;

ii) Alternative explanations stakeholders identify as relevant for the successful use of MDS by care home staff.

Patient and Public Involvement

To keep the person being cared for at the centre of our thinking in ways that inform delivery of care or care home resident benefit, we will convene two care home based resident Patient and Public Involvement (PPI) groups that will meet throughout this review project. A member of this realist review, who is a former carer and IT specialist, will lead the PPI groups. We anticipate that residents and carers input will help us to identify and understand the important contextual factors, and the resource and reasoning that support the implementation of an MDS in long term care settings. The PPI groups input will help us to tailor the stakeholders' consultation, inform our theory(ies) development and ensure that the final refined programme theory(ies) resonates with care home staff and residents' experience.

For peer review only - http://bmjopen.bmj.com/site/about/guidelines.xhtml

DISCUSSION AND CONCLUSION

This realist review will provide a theory-driven understanding of what needs to be in place for the successful implementation an MDS systems in care home settings to benefit residents, staff, families, service managers and commissioners.

Research has demonstrated the value of minimum data sets to commissioners and service providers in the identification of care needs.[41-45] A research study that used care home specific MDS identified specific implementation challenges.[46] It enabled comprehensive analysis of baseline resident data and residents at risk but there was limited staff capacity to support and sustain its completion over time when it was not linked to other data collection responsibilities.[46]

This review addresses a gap in the evidence about how an MDS may be used by different stakeholders to support the direct care of older people resident in care homes.

ETHICS AND DISSEMINATION

The protocol has been submitted to the University of Hertfordshire Ethics Committee. Findings will be disseminated through information exchange with stakeholders, conference presentations, a national consultation on the use of an MDS in English long-term care settings and publications in peer-reviewed journals.

FUNDING

This study/project is funded by the National Institute for Health Research (NIHR) Health Service Research and Delivery programme (HS&DR NIHR127234) and supported by the NIHR Applied Research Collaboration (ARC) East of England.

CONFLICT OF INTEREST

None

For peer review only - http://bmjopen.bmj.com/site/about/guidelines.xhtml

ACKNOWLEDGEMENT

Arne Wolters, Liz Jones and Iain Lang are collaborators on the Developing research resources And minimum data set for Care Homes' Adoption and use (DACHA) study and contributed to early conversations about the review and lead work in the larger study that will use these findings.

DISCLAIMER

The views expressed are those of the author(s) and not necessarily those of the NIHR or the Department of Health and Social Care.

CONTRIBUTORS

Concept and design of the review is imbedded in the Developing research resources And minimum data set for Care Homes' Adoption and use (DACHA) study. SK conducted initial literature search and MKM, GA and CG wrote the first draft of the manuscript. Critical review and refinement of the manuscript was provided by GP, KS, AK, JB, BH, AG, JM, SF, AMT and LI. MKM and CG approved the final version.

For peer review only - http://bmjopen.bmj.com/site/about/guidelines.xhtml

REFERENCES

1. Age UK. Later life in the United Kingdom 2019 2019 [Available from: https://www.ageuk.org.uk/globalassets/age-uk/documents/reports-and-publications/later_life_uk_factsheet.pdf accessed 08/05/2020.
2. Kingston A, Commas-Herrera A, Jagger C. Forecasting the care needs of the older population in England over the next 20 years: estimates from the Population Ageing and Care Simulation (PACSim) modelling study. *Lancet Public Health* 2018;3(9):e447-e55.
3. Kelly A, Kenny C. This POSTnote looks at the numbers of unpaid carers across the UK, and the amount and type of care provided. It examines the impacts of providing unpaid care on carers' education, employment, finances, health and wellbeing, and personal and social relationships. The note reviews the different types of support available for carers and examines evidence on their effectiveness. 2018 [Available from: https://post.parliament.uk/research-briefings/post-pn-0582/ accessed 08/05/2020.
4. Care Quality Commission (CQC). The state of health care and adult social care in England 2016/17: Care Quality Commission (CQC); 2017 [Available from: https://www.cqc.org.uk/sites/default/files/20171011_stateofcare1617_summary.pdf accessed 15/01/2020 2020.
5. Alzheimer's Society. Facts for the media 2019 [Available from: https://www.ageuk.org.uk/globalassets/age-uk/documents/reports-and-publications/later_life_uk_factsheet.pdf accessed 15/01/2020 2020.
6. Musa M, Saga S, Blekken L, et al. The prevalence, incidence, and correlates of faecal incontinence among older people residing in care homes: a systematic review. *Journal of the American Medical Directors Association* 2019;20:956-62.
7. Gordon A, Franklin M, Bradshaw L, et al. Health status of UK care home residents: a cohort study. *Age and Aging* 2014;43(1):97-103.
8. Onder G, Carpenter I, Finne-Soveri H, et al. Assessment of nursing home residents in Europe: the Services and Health for Elderly in Long TERm care (SHELTER) study. *BMC Health Services Research* 2012;12(5):1-10.
9. Booth R. Hundreds of UK care home deaths not added to official coronavirus toll: The Guardian Newspaper 2020 [Available from: https://www.theguardian.com/world/2020/apr/09/covid-19-hundreds-of-uk-care-home-deaths-not-added-to-official-toll accessed 24/04/2020.
10. Age UK. General Election Manifesto 2019: Age UK; 2019 [Available from: https://www.ageuk.org.uk/globalassets/age-uk/documents/campaigns/ge-2019/age-uk-general-election-manifesto-2019.pdf accessed 15/01/2020 2020.
11. Iliffe S, Davies S, Gordon A, et al. Provision of NHS generalist and specialist services to care homes in England: review of surveys. *Primary Health Care Research & Development* 2015;17:122-37.
12. interRAI. he interRAI Organization: Who We Are Mission and Vision 2020 [Available from: https://www.interrai.org/organization/ accessed 03/04/2020.
13. Hutchinson A, Milke D, Malsey S, et al. The resident assessment instrument-minimum data set 2.0 quality indicators: a systematic review. *Health Services Research* 2020;10:1-14.
14. Chen L, Liu L, Peng L, et al. Identifying residents at greater risk for cognitive decline by Minimum Data Set in long-term care settings. *Journal of Clinical Gerontology and Geriatrics* 2014;5(4):122-26.
15. Doupe M, Pass J, J N, et al. How well does the minimum data set measure healthcare use? a validation study. *BMC Health Services Research* 2018;18
16. Gleeny C, Stolee P. Comparing the functional independence measure and the interRAI/MDS for use in the functional assessment of older adults: a review of the literature. *BMC Getriatrics* 2009;9(52):1-12.
17. Hansebo G, Kihlgren M, Ljunggren G, et al. Staff views on the Resident Assessment Instrument, RAI/MDS, in nursing homes, and the use of the Cognitive Performance Scale, CPS, in

For peer review only - http://bmjopen.bmj.com/site/about/guidelines.xhtml

different levels of care in Stockholm, Sweden. *Journal of Advanced Nursing* 1998;28(3):642-53.
18. Wysocki A, Thomas K, Mor V. Functional Improvement Among Short-Stay Nursing Home Residents in the MDS 3.0. *Journal of the American Medical Directors Association* 2015;16(6):470-74.
19. Carpenter G. Accuracy, validity and reliability in assessment and in evaluation of services for older people: the role of the interRAI MDS assessment system. *Age and Aging* 2006;35:327-29.
20. Competition & Marketing Authority (CMA). Care homes market study 2017 [Available from: https://assets.publishing.service.gov.uk/media/5a1fdf30e5274a750b82533a/care-homes-market-study-final-report.pdf accessed 28/02/2020.
21. Gray L, Berg K, Fries B, et al. Sharing clinical information across care settings: the birth of an integrated assessment system. *BMC Health Services Research* 2009;9
22. Jagosh J, Pluye P, Wong G, et al. Critical reflections on realist review: insights from customizing the methodology to the needs of participatory research assessment. *Research Synthesis Methods* 2014;5(2):131-41.
23. Pawson R. Digging for nuggets: how 'bad' research can yield 'good' good evidence. *International Journal of Social Research Methodology* 2006;9(2):127-42.
24. Clark A, Lissel S. Complex critical realism: tenents and application in nursing research. *Advances in Nursing Sciences* 2008;31(4):E67-E79.
25. Rahman A, Applebaum A. The nursing home minimum data set assessment instrument: manifest functions and unintended consequences - past, present, and future. *The Gerontologist* 2009;49(6):727-35.
26. Pawson R. Evidence-based policy: a realist perspective. London: Sage Publications Ltd 2006.
27. Gwyther H, Bobrowicz-Campos E, Luis Alves Apóstolo J, et al. A realist review to understand the efficacy and outcomes of interventions designed to minimise, reverse or prevent the progression of frailty. *Health Psychology Review* 2018;12(4):382-404. doi: 10.1080/17437199.2018.1488601
28. Rycroft-Malone J, Burton CR, Wilkinson J, et al. Collective action for implementation: a realist evaluation of organisational collaboration in healthcare. *Implementation Science* 2016;11(1):17. doi: 10.1186/s13012-016-0380-z
29. May C. Towards a general theory of implementation. *Implementation Science* 2013;8(18):1-14.
30. Jagosh J, Macaulay A, Pierre P, et al. Uncovering the Benefits of ParticipatoryResearch: Implications of a Realist Review forHealth Research and Practice. *The Milbank Quarterly* 2012;90(2):311-46.
31. Wong G, Greenhalgh T, Westhrop G, et al. Development of methodological guidance, publication standards and training materials for realist and meta-narrative reviews: the RAMESES (Realist And Meta-narrative Evidence Syntheses – Evolving Standards) project. *Health Services and Delivery Research* 2014;2
32. Nilsen P. Making sense of implementation theories, models and frameworks. *Implementation Science* 2015;10(53) doi: 10.1186/s13012-015-0242-0
33. Department of Health & Social Care. Policy paper: The future of healthcare: our vision for digital, data and technology in health and care: Department of Health & Social Care; 2018 [Available from: https://www.gov.uk/government/publications/the-future-of-healthcare-our-vision-for-digital-data-and-technology-in-health-and-care accessed 26/03/2020.
34. Hall A, Brown Wilson C, Stanmore E, et al. Implementing monitoring technologies in care homes for people with dementia: A qualitative exploration using Normalization Process Theory. *International Journal of Nursing Studies* 2017;72:60-70.
35. McCormack B, McCance T. Person-centred Nursing: Theory and Practice. Oxford: Wiley Blackwell 2017.

For peer review only - http://bmjopen.bmj.com/site/about/guidelines.xhtml

36. Mitchell G. Person-Centred Care 2015 [Available from: https://blogs.bmj.com/ebn/2015/11/16/person-centred-care/ accessed 30/01/2020 2020.
37. Health Service Executive (HSE). Quality & Risk Management Standard 2007 [Available from: https://www.hse.ie/eng/about/who/oqr009-20080221-v3-quality-and-risk-management-standard.pdf accessed 26/03/2020.
38. Wong G, Greenhalgh T, Westhrop G, et al. Development of methodological guidance, publication standards and training materials for realist and meta-narrative reviews: the RAMESES (Realist And Meta-narrative Evidence Syntheses – Evolving Standards) project. *Health Services and Delivery Research* 2014;2(30)
39. Pawson R, Greenhalgh T, Harvey G, et al. Realist review - a new method of systematic review designed for complex policy interventiions *Journal of Health Services Research & Policy* 2005;10:21-34.
40. Manzano A. The craft of interviewing in realist evaluation. *Evaluation* 2016;22(3):342-60. doi: 10.1177/1356389016638615
41. Carpenter I, Perry M, Challis D, et al. Identification of registered nursing care of residents in English nursing homes using the Minimum Data Set Resident Assessment Instrument (MDS/RAI) and Resource Utilisation Groups version III (RUG-III). *Age and Aging* 2003;32(3):279-85.
42. Hirdes J, Mitchell L, Maxwell C, et al. Beyond the 'iron lungs of gerontology': using evidence to shape the future of nursing homes in Canada. *Canadian Journal on Aging/la Revue canadienne du vieillissement* 2011;30(3):371-90.
43. Jamieson H, Nishtala P, Scrase R, et al. Drug burden and its association with falls among older adults in New Zealand: a national population cross-sectional study. *Drug & Aging* 2018;35(1):73-81.
44. Ogarek J, McCreedy E, Thomas K, et al. Minimum Data Set Changes in Health, End-Stage Disease and Symptoms and Signs Scale: A Revised Measure to Predict Mortality in Nursing Home Residents. *Journal of American Getriatric Society* 2018;66(5):976-81.
45. Szczerbińska K, Hirdes J, Zyczkowska J. Good news and bad news: depressive symptoms decline and undertreatment increases with age in home care and institutional settings. *The American Journal of Geriatric Psychiatry* 2012;20(12):1045-56.
46. Goodman C, Davies S, Gordon A, et al. Optimal NHS service delivery to care homes: a realist evaluation of the features and mechanisms that support effective working for the continuing care of older people in residential settings. *Health Services and Delivery Research* 2017;5(29) doi: 10.3310/hsdr05290

For peer review only - http://bmjopen.bmj.com/site/about/guidelines.xhtml

BMJ Open

The uptake and use of a minimum data set (MDS) for older people living and dying in care homes in England – a realist review protocol

Journal:	*BMJ Open*
Manuscript ID	bmjopen-2020-040397.R1
Article Type:	Protocol
Date Submitted by the Author:	24-Aug-2020
Complete List of Authors:	Musa, Massirfufulay; University of Hertfordshire, Centre for Research in Public Health and Community Care (CRIPACC); King's College London, Faculty of Nursing, Midwifery and Palliative Care<br>Akdur, Gizdem ; University of Hertfordshire, Centre for Research in Public health and Community Care (CRIPACC)<br>Hanratty, Barbara; Newcastle University, Institute of Health and Society / Newcastle University Institute for Ageing<br>Kelly, Sarah; University of Cambridge, Institute of Public Health<br>Gordon, Adam; University of Nottingham, Division of Rehabilitation and Ageing<br>Peryer, Guy; University of East Anglia, Faculty of Medicine and Health Sciences<br>Spilsbury, Karen; University of Leeds, School of Healthcare<br>Killett, Anne; University of East Anglia, Faculty of Medicine and Health Sciences<br>Burton, Jennifer ; University of Glasgow, Institute of Cardiovascular and Medical Sciences<br>Meyer, Julienne; City University of London, National Care Forum<br>Fortescue, Sue; Alzheimer Society Research Network<br>Towers, Ann-Marie; University of Kent, Centre for Health Services Studies<br>Irvine, Lisa; University of Hertfordshire, Centre for Research in Public health and Community Care (CRIPACC)<br>Goodman, Claire; University of Hertfordshire, Centre for Research in Public health and Community Care (CRIPACC)
**Primary Subject Heading**:	Nursing
Secondary Subject Heading:	Public health, Epidemiology, Evidence based practice, Geriatric medicine, Health services research
Keywords:	HEALTH SERVICES ADMINISTRATION & MANAGEMENT, Quality in health care < HEALTH SERVICES ADMINISTRATION & MANAGEMENT, PALLIATIVE CARE, PUBLIC HEALTH, EPIDEMIOLOGY, GERIATRIC MEDICINE

I, the Submitting Author has the right to grant and does grant on behalf of all authors of the Work (as defined in the below author licence), an exclusive licence and/or a non-exclusive licence for contributions from authors who are: i) UK Crown employees; ii) where BMJ has agreed a CC-BY licence shall apply, and/or iii) in accordance with the terms applicable for US Federal Government officers or employees acting as part of their official duties; on a worldwide, perpetual, irrevocable, royalty-free basis to BMJ Publishing Group Ltd ("BMJ") its licensees and where the relevant Journal is co-owned by BMJ to the co-owners of the Journal, to publish the Work in this journal and any other BMJ products and to exploit all rights, as set out in our licence.

The Submitting Author accepts and understands that any supply made under these terms is made by BMJ to the Submitting Author unless you are acting as an employee on behalf of your employer or a postgraduate student of an affiliated institution which is paying any applicable article publishing charge ("APC") for Open Access articles. Where the Submitting Author wishes to make the Work available on an Open Access basis (and intends to pay the relevant APC), the terms of reuse of such Open Access shall be governed by a Creative Commons licence – details of these licences and which Creative Commons licence will apply to this Work are set out in our licence referred to above.

Other than as permitted in any relevant BMJ Author's Self Archiving Policies, I confirm this Work has not been accepted for publication elsewhere, is not being considered for publication elsewhere and does not duplicate material already published. I confirm all authors consent to publication of this Work and authorise the granting of this licence.

For peer review only

Title page

Title:

The uptake and use of a minimum data set (MDS) for older people living and dying in care homes in England – a realist review protocol

Corresponding Author: Claire Goodman

Centre for Research in Public health and Community Care (CRIPACC) University of Hertfordshire AL10 9AB

E-mail: c.goodman@herts.ac.uk

Key words: Care Home, minimum data set, geriatric assessment, nursing tools, older people care

Word count: Abstract (335); main text (3504), references (1409)

Contributing authors: Please insert your contact details

Authors	E-mail addresses	Address
Mr Massirfufulay Kpehe Musa	m.musa2@herts.ac.uk	Centre for Research in Public health and Community Care (CRIPACC) University of Hertfordshire AL10 9AB
Dr Gizdem Akdur	g.akdur@herts.ac.uk	Centre for Research in Public health and Community Care (CRIPACC) University of Hertfordshire AL10 9AB
Professor Barbara Hanratty	barbara.hanratty@newcastle.ac.uk	Population Health Sciences Institute Biomedical Research Building (Second floor) Campus for Ageing and Vitality Newcastle University Newcastle upon Tyne NE4 5PL
Dr Sarah Kelly	sarah.kelly@thisinstitute.cam.ac.uk	Institute of Public Health University of Cambridge CB2 0SR
Dr Adam Gordon	adam.gordon@nottingham.ac.uk	School of Medicine University of Nottingham DE22 3DT
Dr Guy Peryer	g.peryer@uea.ac.uk	Faculty of Medicine and Health Sciences,

For peer review only

For peer review only - http://bmjopen.bmj.com/site/about/guidelines.xhtml

		University of East Anglia, NR4 7TJ
Professor Karen Spilsbury	k.spilsbury@leeds.ac.uk	School of Healthcare, Faculty of Medicine and Health, University of Leeds, Leeds, LS2 9JT
Dr Anne Killett	a.killett@uea.ac.uk	Faculty of Medicine and Health Sciences University of East Anglia Norwich Research Park NR4 7TJ
Dr Jennifer Burton	Jenni.Burton@glasgow.ac.uk	Institute of Cardiovascular and Medical Sciences, University of Glasgow, New Lister Building, Glasgow Royal Infirmary, G31 2ER
Professor Julienne Meyer	J.Meyer@city.ac.uk	National Care Forum
Ms Sue Fortescue	sfortescue@hotmail.co.uk	Alzheimer Society Research Network
Ms Ann-Marie Towers	a.towers@kent.ac.uk	Centre for Health Services Studies University of Kent CT2 7NF
Ms Lisa Irvine	l.irvine@herts.ac.uk	Centre for Research in Public health and Community Care (CRIPACC) University of Hertfordshire AL10 9AB
Professor Claire Goodman	c.goodman@herts.ac.uk	Centre for Research in Public health and Community Care (CRIPACC) University of Hertfordshire AL10 9AB

For peer review only

ABSTRACT

Introduction: Care homes provide nursing and social care for older people who can no longer live independently at home. In the UK, there is no consistent approach to how information about residents' medical history, care needs, and preferences are collected and shared. This limits opportunities to understand the care home population, have a systematic approach to assessment and documentation of care, identify care home residents at risk of deterioration, and review care. Countries with standardised approaches to resident assessment care planning and review (Minimum Data Sets (MDS)) use this data to understand the care home population, guide resource allocation, monitor services delivery and for research. The aim of this realist review is to develop a theory-driven understanding of how care homes staff implement and use MDS to plan and deliver care of individual residents.

Methods and analysis: A realist review will be conducted in three research stages.

Stage one will scope the literature and develop candidate programme theories of what ensures effective uptake and sustained implementation of an MDS.

Stage two will test and refine these theories through further iterative searches of the evidence from the literature to establish how effective uptake of an MDS can be achieved.

Stage three will consult with relevant stakeholders to test or refine the programme theory (theories) of how an MDS works at the resident level of care for different stakeholders and in what circumstances. Data synthesis will use realist logic of analysis to align data from each eligible article with possible context-mechanism-outcome (CMO) configurations, or specific elements that answer the research questions.

Ethics and dissemination: The University of Hertfordshire Ethics Committee has approved this study (HSK/SF/UH/04169). Findings will be disseminated through briefings with stakeholders, conference presentations, a national consultation on the use of an MDS in UK long-term care settings, publications in peer-reviewed journals, and in print and social media publications accessible to residents, relatives, and care home staff.

Review registration number: It is registered on the International Prospective Register of Systematic Reviews (PROSPERO) - CRD42020171323 (registered 09/03/2020).

Keywords: Care Home, minimum data set, geriatric assessment, nursing tools, older people care

For peer review only - http://bmjopen.bmj.com/site/about/guidelines.xhtml

Strengths and limitations of this study

- The review will identify what needs to be in place to support the implementation of an MDS in long term care settings where standardised approaches to resident assessment and data collection are not in place
- The review will demonstrate how using an MDS affects the everyday work and care practices of staff and its impact on residents' care
- The synthesis will integrate qualitative and quantitative evidence that offers transferable learning for long term care settings that do not currently use an MDS
- There are time constraints that may result in the team focusing on or prioritising some aspects of an MDS implementation over others

BACKGROUND

There are nearly 12 million (11,989,322) people aged 65 years and above in the United Kingdom (UK) of which an estimated 1.6 million are aged 85 years and above and more than 500,000 (579,776) people are aged 90 years and above.[1] However, with greater longevity (e.g. age 85 years and above) comes higher levels of dependency, dementia and comorbidity,[2] which in turn intensify the need for social care services.[3] Approximately 420,000 older people in England and Wales live in care homes. [4] Care home is a generic term that refers to facilities where a number of older people live together and have staff available 24 hours to provide personal care (e.g. residential care or assisted living/supportive housing facilities), and those facilities where a qualified nurse is required on duty 24 hours to provide additional nursing care for more dependent residents (e.g. nursing homes or skilled nursing facilities).[5] The care home population represents the oldest and most vulnerable group of older people,[6] with approximately 70% of them living with cognitive impairment, [6 7] and 76% requiring assistance with mobility.[8]

In the UK, there is no consistent approach to how information about residents' medical history, care needs, and preferences is collected and used. The absence of a national mandate, lack of links with National Health Services (NHS) data, and implementation challenges have meant that a minimum data set (MDS) and data-driven approaches to resident assessment have been limited to single projects.[9] The lack of a link between care home data and the NHS data recently became evident when figures reported by the Office for National Statistics during the first three weeks of Covid-19 underestimated the impact of the pandemic among care home residents.[10] All care homes, however, routinely collect

For peer review only - http://bmjopen.bmj.com/site/about/guidelines.xhtml

large amounts of data about their residents. The challenge is to establish systems of assessment and recording that are evidence-based, accessible and valuable to those using, providing, commissioning and regulating care services. Without a unified record there may be duplication of assessments, communication failures and, unmet care needs.[11 12] Over the next two decades the number of older people likely to need long term care will increase;[2] so determining consistent ways to assess and document care for residents in these settings is a priority.

A minimum data set (MDS) in this realist review is defined as a comprehensive, standardised account of the characteristics and needs and ongoing care of residents living in long-term care settings. The review concentrates on how an MDS is used by care home staff and what supports effective uptake for the benefit of individual residents. It also takes account of the involvement of residents themselves and their family in influencing how an MDS is used.

There are multiple versions of MDS, which are often country specific. However, all versions of MDS share a common language and are designed to support an integrated system of care that can support cross-sector clinical and managerial decision-making. One example of an MDS is the International Resident Assessment Instrument (InterRAI), developed for long term care facilities (LTCF).[13] The use of an MDS is often mandated and/or linked to national reimbursement systems and quality monitoring. Research has demonstrated the value of an MDS to commissioners and service providers in enabling identification of care needs and residents at risk of ill health.[14-18] They can provide a comprehensive account of resident characteristics, resource use, and care outcomes in key areas (e.g. activities of daily living, cognitive performance, pain, cost of care, and infection).[19]For long term care settings making the transition to standardised approaches to data collection, little is known about what needs to be in place to implement an MDS and how its use impacts on staff work, time away from care, knowledge of the care home residents, working with other health care professionals and benefits (or not) to residents, staff and residents' families.

METHODS AND ANALYSIS

Review aim and objectives

Aim

For peer review only - http://bmjopen.bmj.com/site/about/guidelines.xhtml

To develop a theory-driven understanding of how care homes' staff can effectively implement and use MDS to plan and deliver care of individual residents.

Objectives

1. Develop a programme theory describing contexts that can support the uptake and use of an MDS in care homes.
2. Identify in what circumstances the use of an MDS produces improved outcomes (including resource use) for an individual resident, their family, and the care home staff and their employing organisation.

Study design

We will conduct a realist review which seeks to formulate, test and refine the programme theory while assessing whether and how the programme succeeds in the local setting,[20] in order to generate important insights for the United Kingdom. A programme theory is an overarching theory or model of how an intervention is expected to work[21] and it helps to explain (some of) 'how and why, in the "real world", a specific programme "works", for whom, to what extent and in which contexts'.[22] The unit of analysis in a realist review is the ideas and assumptions (i.e., the programme theories) that underlie an intervention and explain how it works to achieve the desired outcomes.

A realist review is an interpretive, theory-driven approach to evidence synthesis[23 24] to develop a programme theory of how an intervention is expected to work. Interventions like the minimum data set (MDS) will always rely on human agency to affect change. A realist approach argues that the features or elements of the intervention will produce a range of potential responses to the intervention which will impact on the outcomes.[20 23] It assumes that there is a knowable, independent reality that will shape how different participants react to an intervention, whether they are aware of these influences or not.[25] Thus, uptake and implementation of an MDS can lead to different outcomes for different stakeholders (e.g. residents and their relatives, staff, commissioners, regulators) depending on who is involved, the resources available and how the MDS is used.[26 27]

Using a realist approach,[20] there are the four key linked concepts for explaining and building a theory of how a programme works: (i) contexts (C), which are often the 'backdrop' of interventions;[23] (ii) mechanisms (M), which are not observed directly but account for what it is about programmes that make them work,[28 29] characterised as *"a process that bring about or prevents some change in a concrete system"*[30] (iii) outcomes (O) of the intervention (planned or unplanned, visible or not) or

For peer review only - http://bmjopen.bmj.com/site/about/guidelines.xhtml

strategies of the intervention;[31] It is the Context-Mechanism-Outcome (CMO) configurations (models indicating how programmes activate mechanisms for whom and in what conditions, to elicit outcomes) that are the building blocks of the theory.[24] Thus, in care home settings, staff understanding of their responsibility for completing an MDS could be a context (C), which triggers how staff prioritise recording information as part of care work (M) to identify residents at risk of deterioration (O).

This review will follow Realist And Meta-narrative Evidence Syntheses: Evolving Standards (RAMESES) guidance.[32] Organised in three stages, we will first undertake a scoping of the literature to identify care home specific work on the uptake of MDS and develop relevant theories around staff uptake and implementation and outcomes specific to the use of an MDS in long term (care home) settings. Stage two will test and refine the emergent theories that underpin the use of an MDS and that leads to both intended and unintended outcomes for staff and residents. Stage three will synthesise the findings to establish how and when the use of an MDS achieves different outcomes for residents, families, staff and organisations.

Stages of the review process

Stage 1: Defining the scope of the review, identifying existing theories and theory development

This review is nested within a larger review (“*A systematic review of process and contextual factors that influence research implementation in care homes and identification of key measures and outcomes in care home research*”; PROSPERO reference: CRD42020155923). The literature identified from the larger review is the starting point for the scoping work. Search results relevant to MDS will be downloaded into Covidence software. Two reviewers (MKM and GA) will independently screen titles and abstracts identified by electronic search and applied the selection criteria to potentially relevant full-text papers.[33] Disagreement between MKM and GA will be resolved by the third reviewer, CG.

Based on earlier work that used an MDS to collect data and cross team discussions,[34] the initial programme theory will focus on how an MDS is used in long term care. This will be the basis for scoping the literature on the challenges of changing systems of care, the need for a policy or regulator

For peer review only - http://bmjopen.bmj.com/site/about/guidelines.xhtml

mandate, how it affects patterns of working in the care home, staff involvement in data entry and changes residents care. At this stage we are not assuming causality but recognise that these are likely to influence uptake and use and resident and staff outcomes. Studies that have used MDS, or similar approaches to document resident, staff and organisational outcomes but do not address questions of implementation and use will be reviewed to identify supplementary evidence on related issues of interest (e.g. accuracy of data, time commitment and how information was used and by who). The search strategy will include citation searching and grey literature, and will be iteratively extended and refocused as the review progresses.

We will develop a series of 'if-then statements' to summarise the dominant arguments and supporting evidence of what supports the uptake and use of MDS in long-term care settings. This will inform the development of hypotheses that posit possible contexts (C) that are the backdrop to successful (or not) uptake[23] of MDS; the mechanisms (M) they trigger[30] and planned or unplanned Outcomes (O) arising from the use of MDS.[31] Possible context-mechanism-outcome (CMO) configurations will be discussed across the research team and with subject experts on MDS and will inform stage two of the review phase and additional searches.

There are several ways to conceptualise the development and uptake of MDS as many have their roots in medical approaches to assessment and health systems design. It is therefore likely that the review will be informed by and aim to build on theories of implementation in long-term care,[30 35] uptake of technological innovation[36 37] assessment of older people with complex needs,[2 4 8] person-centred care,[38 39] and risk management and quality assurance.[40]

The introduction of an MDS in care homes is sensitive to the resource and policy constraints under which the care homes operate. Candidate theories will therefore consider the role of the regulator and legal frameworks that incentivise (or not) data sharing across organisations.

The scoping of the literature will focus on studies that report on how an MDS is used in long term care settings. Outcomes of interest will be established by the project team as an iterative process but are likely to include evidence of its impact on: accuracy of reporting, needs assessment, staff workload, quality of care, resource use, staff satisfaction and access to care. No geographical restrictions will

For peer review only - http://bmjopen.bmj.com/site/about/guidelines.xhtml

apply, although we will only include studies that are published in English and focus on the uptake and use of an MDS in long term care settings.

Literature selection, quality appraisal and data extraction criteria

There will be no restriction on the types of study design for eligibility.[41] Article selection will be based on the extent to which research on the uptake and routine use of an MDS can contribute to the development of a programme theory of implementation of MDS in long term care settings.

Included studies are likely to cover the following:

- Studies on the introduction and development of an MDS with care home staff
- Studies that focus on the inclusion and engagement of care home staff, residents and their representatives in sharing resident data with the specific remit of improving resident outcomes
- Implementation studies that provide evidence on what facilitates and inhibits the shared documentation and care planning in care home setting including digital innovation
- Studies on commissioning services for care homes based on care home generated data

Screening and selection of articles will take place in two stages (title and abstract, and full text).[42] The methodological quality of included papers will be carried out in accordance with previous appraisal work within a realist project.[27 42] Two reviewers (MKM and GA) will independently screen 10 articles and cross-check results to discuss emergent ideas and themes and establish consensus on the relevance of the documents. Any disagreement resolved with ongoing discussions with the wider team.

Quality appraisal of included articles

The quality appraisal of included studies will be combined with data extraction, a technique usually employed in realist review.[43] Realist reviews employ various techniques to assess the quality of evidence by drawing on evidence from a wider range of sources unlike traditional systematic reviews that only focus on the methodological quality of studies.[27 42] As quality of evidence is not limited to

For peer review only - http://bmjopen.bmj.com/site/about/guidelines.xhtml

the methodological quality, or hierarchy of evidence in realist reviews,[27] each article in this review will be assessed based on its trustworthiness and applicability to the research questions. Consistent with the realist approach, we will use an iterative approach to determine whether an article is considered "*good enough and relevant*" to answer our research questions.[44] Good enough will be based on the reviewers' own assessment of the quality of evidence, for example if it is considered to be of a sufficient standard for the research question, and relevance will relate to whether the authors provided sufficient descriptive detail and/or theoretical discussion to contribute to the initial programme theories development.[33]

The quality appraisal in this review will be assessed on a case by case basis considering the opportunities for learning, scientific rigor of evidence and relevance to the review questions.[43] Two reviewers (MKM and GA), in consultation with CG will lead this process. Weaker papers and those with equivocal or negative findings will be considered if they contribute to the overall programme theories.

Data extraction

Data extraction will be conducted on the basis of relevance to the review questions and will be based on realist guidelines to address questions that explore "what is it that supports (or hinders) an MDS implementation in care homes, and how care home staff use and interpret an MDS to guide residents' care?"[42] From the extracted data, two reviewers (MM and GA) will independently rate the studies as either yes, no, or maybe in terms of whether the particular article meets inclusion criteria. We will use "maybe" for issues that cannot be answered based on the information available in the publication. Then the two reviewers (MM and GA) will meet with a third reviewer (CG) who will serve as an adjudicator to verify, confirm, or reject inclusion of the data. From relevant articles, several 'if-then' statements will be made from which initial programme theories will be made.

Data synthesis

Data synthesis of the scoping phase will use realist logic of analysis to align data from each eligible article with possible context-mechanism-outcome (CMO) configurations,[20] or specific elements that answer the research questions. These emerging findings, and putative patterns of association within the data will be tested further in stage two, to build causal explanations based on the observed interactions between context, mechanism, and outcomes.

For peer review only - http://bmjopen.bmj.com/site/about/guidelines.xhtml

Stage 2: Testing and refining the programme theories

Further iterative searches of the evidence will be directly informed by the CMOs developed in stage one as candidate programme theories. The iterative circle will continue throughout the course of the review until theoretical saturation has been achieved. [20 32]

Data will be extracted using a bespoke data extraction form. It will include descriptive data on study characteristics and is likely to focus on what can be learnt about the role and work of staff, the resources required to implement an MDS, the features of the settings (e.g., workforce capacity, size of care homes), explicit and implicit theories for how interventions were anticipated to work, and patient and carer outcomes. A sample of the papers, including those that appear to offer most learning and their completed data extraction forms will be shared across the project team to support ongoing discussion and debate of the candidate theory(ies) and their supporting evidence.

Stage 3: Analysis and synthesis of evidence from the proposed programme theories

To support hypothesis refinement and "fine tune" the theory(ies) that show the most promise, we will further test findings from stage two in a series of interviews.[45] We will do this through stakeholders' consultation (Box 1). Four group interviews with frontline staff will take place including care home staff, managers, including staff from care homes who use predominately paper based records and staff who routinely use electronic records for their residents. We will also carry out individual semi-structured interviews with up to eight stakeholders who are experts regarding the use of care home residents' data.

Stakeholders' selection will be purposive based on their knowledge of using MDS in and with care homes. At each stage of the review we will present and discuss the programme theories with the supporting evidence for discussion with the whole research project team.

Box 1: Stakeholders' consultation

During stakeholders' consultation interviews, we will explore:

i) The fit between the emerging programme theories and how stakeholders understand what is needed for the development and use of MDS in long term care settings
ii) Alternative explanations stakeholders identify as relevant for the successful use of MDS by care home staff

For peer review only

A summary of the review process is presented in Figure 1. The double arrows within or between stages indicate iterative processes of the review.

Figure 1: The Realist Review Processes

Patient and Public Involvement

To keep the person being cared for at the centre of our thinking in ways that inform delivery of care or care home resident benefit, we will convene two care home based resident Patient and Public Involvement (PPI) groups that will meet throughout this review project. A member of this realist review, who is a former carer and IT specialist, will lead the PPI groups. We anticipate that input from residents and carers will help us to identify and understand the important contextual factors, and the resource and reasoning that support the implementation of an MDS in long term care settings. The PPI groups input will help us to tailor the stakeholders' consultation, inform our theory (or theories) development and ensure that the final refined programme theory(ies) resonates with care home staff and residents' experience.

DISCUSSION AND CONCLUSION

This realist review will provide a theory-driven understanding of what needs to be in place for the successful implementation an MDS systems in care home settings to benefit residents, staff, families, service managers and commissioners.

Research has demonstrated the value of minimum data sets to commissioners and service providers in the identification of care needs.[46-50] A research study that used care home specific MDS identified specific implementation challenges.[34] It enabled comprehensive analysis of baseline resident data and residents at risk but there was limited staff capacity to support and sustain its completion over time when it was not linked to other data collection responsibilities.[34] This review addresses a gap in the evidence about what is needed to support uptake and implementation of an MDS, what needs to be in place for effective uptake and how an MDS is used in different circumstances to enable key care outcomes for residents. By identifying the causal mechanisms at work the review findings will directly

For peer review only - http://bmjopen.bmj.com/site/about/guidelines.xhtml

inform decision-making about how to design, tailor and implement an MDS that is acceptable to staff and can inform residents' everyday care.

ETHICS AND DISSEMINATION

The University of Hertfordshire Ethics Committee has approved this study (HSK/SF/UH/04169). Findings will be disseminated through briefings with stakeholders, conference presentations, a national consultation on the use of an MDS in UK long-term care settings, and publications in peer-reviewed journals, and in print and social media publications accessible to residents, relatives, and care home staff.

FUNDING

This study/project is funded by the National Institute for Health Research (NIHR) Health Service Research and Delivery programme (HS&DR NIHR127234) and supported by the NIHR Applied Research Collaboration (ARC) East of England. This review started from December 2019 and it will end by 31st December 2020.

CONFLICT OF INTEREST

None

ACKNOWLEDGEMENT

Arne Wolters, Liz Jones and Iain Lang are collaborators on the Developing research resources And minimum data set for Care Homes' Adoption and use (DACHA) study and contributed to early conversations about the review and lead work in the larger study that will use these findings. We thank the reviewers Catherine Powell and Matthias Hoben whose constructive comments/suggestions helped to improve and clarify this manuscript.

For peer review only - http://bmjopen.bmj.com/site/about/guidelines.xhtml

DISCLAIMER

The views expressed are those of the author(s) and not necessarily those of the NIHR or the Department of Health and Social Care.

CONTRIBUTORS

Concept and design of the review are embedded in the Developing research resources And minimum data set for Care Homes' Adoption and use (DACHA) study. SK conducted the initial literature search and MKM, GA and CG wrote the first draft of the manuscript. Critical review and refinement of the manuscript was provided by GP, KS, AK, JB, BH, AG, JM, SF, AMT and LI. MKM, GA and CG approved the final version.

For peer review only - http://bmjopen.bmj.com/site/about/guidelines.xhtml

REFERENCES

1. Age UK. Later life in the United Kingdom 2019 2019 [Available from: https://www.ageuk.org.uk/globalassets/age-uk/documents/reports-and-publications/later_life_uk_factsheet.pdf accessed 08/05/2020.
2. Kingston A, Commas-Herrera A, Jagger C. Forecasting the care needs of the older population in England over the next 20 years: estimates from the Population Ageing and Care Simulation (PACSim) modelling study. *Lancet Public Health* 2018;3(9):e447-e55.
3. Kelly A, Kenny C. This POSTnote looks at the numbers of unpaid carers across the UK, and the amount and type of care provided. It examines the impacts of providing unpaid care on carers' education, employment, finances, health and wellbeing, and personal and social relationships. The note reviews the different types of support available for carers and examines evidence on their effectiveness. 2018 [Available from: https://post.parliament.uk/research-briefings/post-pn-0582/ accessed 08/05/2020.
4. Care Quality Commission (CQC). The state of health care and adult social care in England 2016/17: Care Quality Commission (CQC); 2017 [Available from: https://www.cqc.org.uk/sites/default/files/20171011_stateofcare1617_summary.pdf accessed 15/01/2020 2020.
5. Underwood M, Lamb SE, Eldridge S, et al. Exercise for depression in care home residents: a randomised controlled trial with cost-effectiveness analysis (OPERA). *Health technology assessment (Winchester, England)* 2013;17(18):1-281. doi: 10.3310/hta17180 [published Online First: 2013/05/02]
6. Musa M, Saga S, Blekken L, et al. The prevalence, incidence, and correlates of faecal incontinence among older people residing in care homes: a systematic review. *Journal of the American Medical Directors Association* 2019;20:956-62.
7. Alzheimer's Society. Facts for the media 2019 [Available from: https://www.ageuk.org.uk/globalassets/age-uk/documents/reports-and-publications/later_life_uk_factsheet.pdf accessed 15/01/2020 2020.
8. Gordon A, Franklin M, Bradshaw L, et al. Health status of UK care home residents: a cohort study. *Age and Aging* 2014;43(1):97-103.
9. Onder G, Carpenter I, Finne-Soveri H, et al. Assessment of nursing home residents in Europe: the Services and Health for Elderly in Long TERm care (SHELTER) study. *BMC Health Services Research* 2012;12(5):1-10.
10. Booth R. Hundreds of UK care home deaths not added to official coronavirus toll: The Guardian Newspaper 2020 [Available from: https://www.theguardian.com/world/2020/apr/09/covid-19-hundreds-of-uk-care-home-deaths-not-added-to-official-toll accessed 24/04/2020.
11. Age UK. General Election Manifesto 2019: Age UK; 2019 [Available from: https://www.ageuk.org.uk/globalassets/age-uk/documents/campaigns/ge-2019/age-uk-general-election-manifesto-2019.pdf accessed 15/01/2020 2020.
12. Iliffe S, Davies S, Gordon A, et al. Provision of NHS generalist and specialist services to care homes in England: review of surveys. *Primary Health Care Research & Development* 2015;17:122-37.
13. Hutchinson A, Milke D, Malsey S, et al. The resident assessment instrument-minimum data set 2.0 quality indicators: a systematic review. *Health Services Research* 2020;10:1-14.
14. Chen L, Liu L, Peng L, et al. Identifying residents at greater risk for cognitive decline by Minimum Data Set in long-term care settings. *Journal of Clinical Gerontology and Geriatrics* 2014;5(4):122-26.

For peer review only - http://bmjopen.bmj.com/site/about/guidelines.xhtml

15. Doupe M, Pass J, J N, et al. How well does the minimum data set measure healthcare use? a validation study. *BMC Health Services Research* 2018;18
16. Gleeny C, Stolee P. Comparing the functional independence measure and the interRAI/MDS for use in the functional assessment of older adults: a review of the literature. *BMC Getriatrics* 2009;9(52):1-12.
17. Hansebo G, Kihlgren M, Ljunggren G, et al. Staff views on the Resident Assessment Instrument, RAI/MDS, in nursing homes, and the use of the Cognitive Performance Scale, CPS, in different levels of care in Stockholm, Sweden. *Journal of Advanced Nursing* 1998;28(3):642-53.
18. Wysocki A, Thomas K, Mor V. Functional Improvement Among Short-Stay Nursing Home Residents in the MDS 3.0. *Journal of the American Medical Directors Association* 2015;16(6):470-74.
19. Carpenter G. Accuracy, validity and reliability in assessment and in evaluation of services for older people: the role of the interRAI MDS assessment system. *Age and Aging* 2006;35:327-29.
20. Pawson R, Greenhalgh T, Harvey G, et al. Realist review - a new method of systematic review designed for complex policy interventiions *Journal of Health Services Research & Policy* 2005;10:21-34.
21. Maden M, Cunliffe A, McMahon N, et al. Use of programme theory to understand the differential effects of interventions across socio-economic groups in systematic reviews—a systematic methodology review. *Systematic Reviews* 2017;6(1):266. doi: 10.1186/s13643-017-0638-9
22. Wong G, Westhorp G, Manzano A, et al. RAMESES II reporting standards for realist evaluations. *BMC Medicine* 2016;14(1):96. doi: 10.1186/s12916-016-0643-1
23. Jagosh J, Pluye P, Wong G, et al. Critical reflections on realist review: insights from customizing the methodology to the needs of participatory research assessment. *Research Synthesis Methods* 2014;5(2):131-41.
24. Pawson R. Digging for nuggets: how 'bad' research can yield 'good' good evidence. *International Journal of Social Research Methodology* 2006;9(2):127-42.
25. Clark A, Lissel S. Complex critical realism: tenents and application in nursing research. *Advances in Nursing Sciences* 2008;31(4):E67-E79.
26. Rahman A, Applebaum A. The nursing home minimum data set assessment instrument: manifest functions and unintended consequences - past, present, and future. *The Gerontologist* 2009;49(6):727-35.
27. Pawson R. Evidence-based policy: a realist perspective. London: Sage Publications Ltd 2006.
28. Gwyther H, Bobrowicz-Campos E, Luis Alves Apóstolo J, et al. A realist review to understand the efficacy and outcomes of interventions designed to minimise, reverse or prevent the progression of frailty. *Health Psychology Review* 2018;12(4):382-404. doi: 10.1080/17437199.2018.1488601
29. Rycroft-Malone J, Burton CR, Wilkinson J, et al. Collective action for implementation: a realist evaluation of organisational collaboration in healthcare. *Implementation Science* 2016;11(1):17. doi: 10.1186/s13012-016-0380-z
30. May C. Towards a general theory of implementation. *Implementation Science* 2013;8(18):1-14.
31. Jagosh J, Macaulay A, Pierre P, et al. Uncovering the Benefits of ParticipatoryResearch: Implications of a Realist Review forHealth Research and Practice. *The Milbank Quarterly* 2012;90(2):311-46.
32. Wong G, Greenhalgh T, Westhrop G, et al. Development of methodological guidance, publication standards and training materials for realist and meta-narrative reviews: the RAMESES (Realist And Meta-narrative Evidence Syntheses – Evolving Standards) project. *Health Services and Delivery Research* 2014;2

For peer review only - http://bmjopen.bmj.com/site/about/guidelines.xhtml

33. Bunn F, Goodman C, Russell B, et al. Supporting shared decision making for older people with multiple health and social care needs: a realist synthesis. *BMC Geriatrics* 2018;18(1):165. doi: 10.1186/s12877-018-0853-9
34. Goodman C, Davies S, Gordon A, et al. Optimal NHS service delivery to care homes: a realist evaluation of the features and mechanisms that support effective working for the continuing care of older people in residential settings. *Health Services and Delivery Research* 2017;5(29) doi: 10.3310/hsdr05290
35. Nilsen P. Making sense of implementation theories, models and frameworks. *Implementation Science* 2015;10(53) doi: 10.1186/s13012-015-0242-0
36. Department of Health & Social Care. Policy paper: The future of healthcare: our vision for digital, data and technology in health and care: Department of Health & Social Care; 2018 [Available from: https://www.gov.uk/government/publications/the-future-of-healthcare-our-vision-for-digital-data-and-technology-in-health-and-care accessed 26/03/2020.
37. Hall A, Brown Wilson C, Stanmore E, et al. Implementing monitoring technologies in care homes for people with dementia: A qualitative exploration using Normalization Process Theory. *International Journal of Nursing Studies* 2017;72:60-70.
38. McCormack B, McCance T. Person-centred Nursing: Theory and Practice. Oxford: Wiley Blackwell 2017.
39. Mitchell G. Person-Centred Care 2015 [Available from: https://blogs.bmj.com/ebn/2015/11/16/person-centred-care/ accessed 30/01/2020 2020.
40. Health Service Executive (HSE). Quality & Risk Management Standard 2007 [Available from: https://www.hse.ie/eng/about/who/oqr009-20080221-v3-quality-and-risk-management-standard.pdf accessed 26/03/2020.
41. Wong G, Greenhalgh T, Westhrop G, et al. Development of methodological guidance, publication standards and training materials for realist and meta-narrative reviews: the RAMESES (Realist And Meta-narrative Evidence Syntheses – Evolving Standards) project. *Health Services and Delivery Research* 2014;2(30)
42. Abayneh S, Lempp H, Manthorpe J, et al. Development of programme theory for integration of service user and caregiver involvement in mental health system strengthening: protocol for realist systematic review. *Int J Ment Health Syst* 2018;12:41-41. doi: 10.1186/s13033-018-0220-4
43. Greenhalgh J, Pawson R, Wright J, et al. Functionality and feedback: a protocol for a realist synthesis of the collation, interpretation and utilisation of PROMs data to improve patient care. *BMJ Open* 2014;4(7):e005601. doi: 10.1136/bmjopen-2014-005601
44. Rycroft-Malone J, Burton C, Hall B, et al. Improving skills and care standards in the support workforce for older people: a realist review. *BMJ Open* 2014;4(5):e005356. doi: 10.1136/bmjopen-2014-005356
45. Manzano A. The craft of interviewing in realist evaluation. *Evaluation* 2016;22(3):342-60. doi: 10.1177/1356389016638615
46. Carpenter I, Perry M, Challis D, et al. Identification of registered nursing care of residents in English nursing homes using the Minimum Data Set Resident Assessment Instrument (MDS/RAI) and Resource Utilisation Groups version III (RUG-III). *Age and Aging* 2003;32(3):279-85.
47. Hirdes J, Mitchell L, Maxwell C, et al. Beyond the 'iron lungs of gerontology': using evidence to shape the future of nursing homes in Canada. *Canadian Journal on Aging/la Revue canadienne du vieillissement* 2011;30(3):371-90.
48. Jamieson H, Nishtala P, Scrase R, et al. Drug burden and its association with falls among older adults in New Zealand: a national population cross-sectional study. *Drug & Aging* 2018;35(1):73-81.

For peer review only

For peer review only - http://bmjopen.bmj.com/site/about/guidelines.xhtml

49. Ogarek J, McCreedy E, Thomas K, et al. Minimum Data Set Changes in Health, End-Stage Disease and Symptoms and Signs Scale: A Revised Measure to Predict Mortality in Nursing Home Residents. *Journal of American Getriatric Society* 2018;66(5):976-81.

50. Szczerbińska K, Hirdes J, Zyczkowska J. Good news and bad news: depressive symptoms decline and undertreatment increases with age in home care and institutional settings. *The American Journal of Geriatric Psychiatry* 2012;20(12):1045-56.

For peer review only

For peer review only - http://bmjopen.bmj.com/site/about/guidelines.xhtml

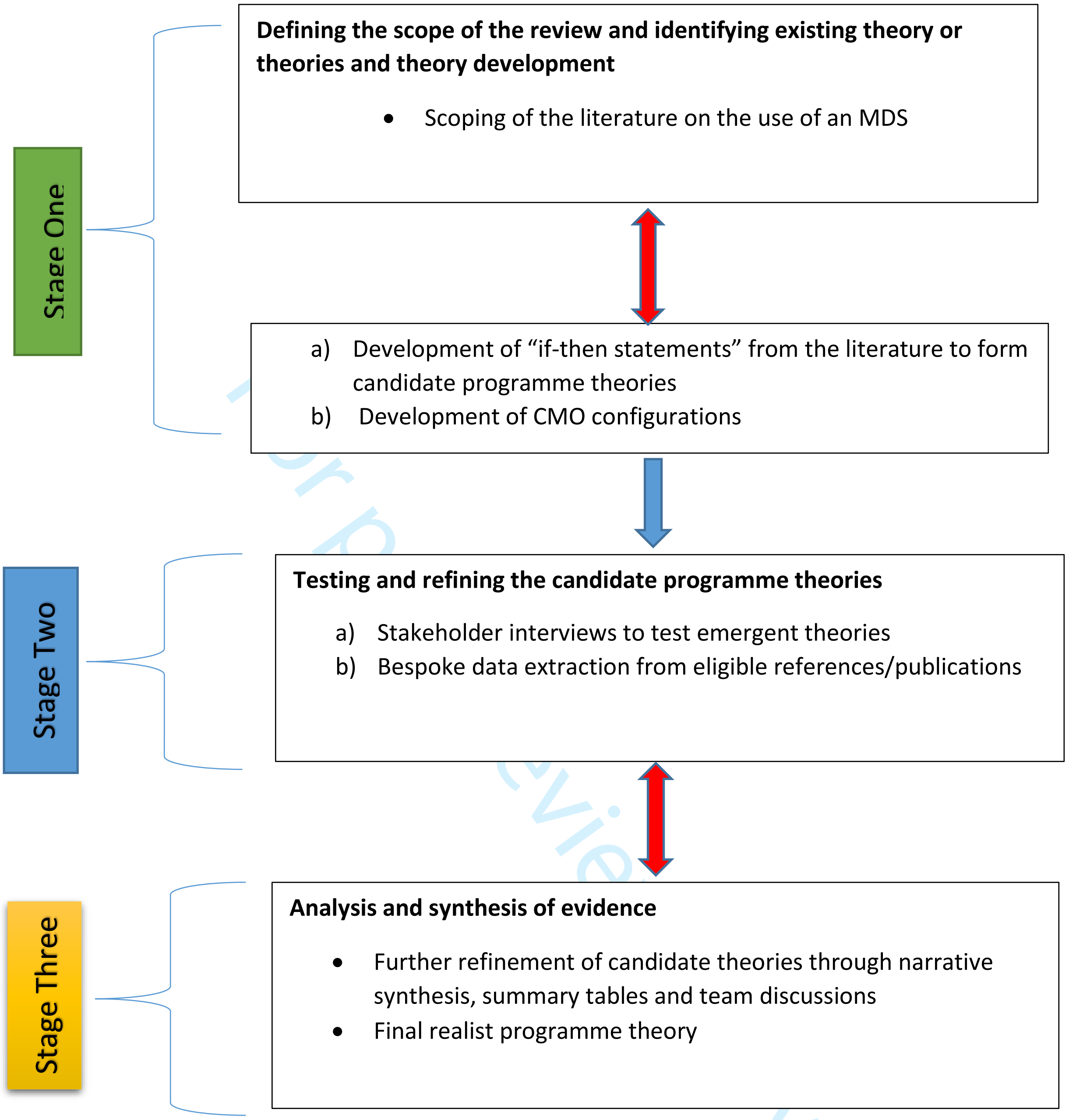
Stage One
Defining the scope of the review and identifying existing theory or theories and theory development
Scoping of the literature on the use of an MDS
a) Development of "if-then statements" from the literature to form candidate programme theories
b) Development of CMO configurations
Stage Two
Testing and refining the candidate programme theories
a) Stakeholder interviews to test emergent theories
b) Bespoke data extraction from eligible references/publications
Stage Three
Analysis and synthesis of evidence
Further refinement of candidate theories through narrative synthesis, summary tables and team discussions
Final realist programme theory

BMJ Open

The uptake and use of a minimum data set (MDS) for older people living and dying in care homes in England – a realist review protocol

Journal:	*BMJ Open*
Manuscript ID	bmjopen-2020-040397.R2
Article Type:	Protocol
Date Submitted by the Author:	15-Sep-2020
Complete List of Authors:	Musa, Massirfufulay; University of Hertfordshire, Centre for Research in Public Health and Community Care (CRIPACC); King's College London, Faculty of Nursing, Midwifery and Palliative Care<br>Akdur, Gizdem ; University of Hertfordshire, Centre for Research in Public health and Community Care (CRIPACC)<br>Hanratty, Barbara; Newcastle University, Institute of Health and Society / Newcastle University Institute for Ageing<br>Kelly, Sarah; University of Cambridge, Institute of Public Health<br>Gordon, Adam; University of Nottingham, Division of Rehabilitation and Ageing<br>Peryer, Guy; University of East Anglia, Faculty of Medicine and Health Sciences<br>Spilsbury, Karen; University of Leeds, School of Healthcare<br>Killett, Anne; University of East Anglia, Faculty of Medicine and Health Sciences<br>Burton, Jennifer ; University of Glasgow, Institute of Cardiovascular and Medical Sciences<br>Meyer, Julienne; City University of London, National Care Forum<br>Fortescue, Sue; Alzheimer Society Research Network<br>Towers, Ann-Marie; University of Kent, Centre for Health Services Studies<br>Irvine, Lisa; University of Hertfordshire, Centre for Research in Public health and Community Care (CRIPACC)<br>Goodman, Claire; University of Hertfordshire, Centre for Research in Public health and Community Care (CRIPACC)
**Primary Subject Heading**:	Nursing
Secondary Subject Heading:	Public health, Epidemiology, Evidence based practice, Geriatric medicine, Health services research
Keywords:	HEALTH SERVICES ADMINISTRATION & MANAGEMENT, Quality in health care < HEALTH SERVICES ADMINISTRATION & MANAGEMENT, PALLIATIVE CARE, PUBLIC HEALTH, EPIDEMIOLOGY, GERIATRIC MEDICINE

I, the Submitting Author has the right to grant and does grant on behalf of all authors of the Work (as defined in the below author licence), an exclusive licence and/or a non-exclusive licence for contributions from authors who are: i) UK Crown employees; ii) where BMJ has agreed a CC-BY licence shall apply, and/or iii) in accordance with the terms applicable for US Federal Government officers or employees acting as part of their official duties; on a worldwide, perpetual, irrevocable, royalty-free basis to BMJ Publishing Group Ltd ("BMJ") its licensees and where the relevant Journal is co-owned by BMJ to the co-owners of the Journal, to publish the Work in this journal and any other BMJ products and to exploit all rights, as set out in our licence.

The Submitting Author accepts and understands that any supply made under these terms is made by BMJ to the Submitting Author unless you are acting as an employee on behalf of your employer or a postgraduate student of an affiliated institution which is paying any applicable article publishing charge ("APC") for Open Access articles. Where the Submitting Author wishes to make the Work available on an Open Access basis (and intends to pay the relevant APC), the terms of reuse of such Open Access shall be governed by a Creative Commons licence – details of these licences and which Creative Commons licence will apply to this Work are set out in our licence referred to above.

Other than as permitted in any relevant BMJ Author's Self Archiving Policies, I confirm this Work has not been accepted for publication elsewhere, is not being considered for publication elsewhere and does not duplicate material already published. I confirm all authors consent to publication of this Work and authorise the granting of this licence.

For peer review only

Title page

Title:

The uptake and use of a minimum data set (MDS) for older people living and dying in care homes in England – a realist review protocol

Corresponding Author: Claire Goodman

Centre for Research in Public health and Community Care (CRIPACC) University of Hertfordshire AL10 9AB

E-mail: c.goodman@herts.ac.uk

Key words: Care Home, minimum data set, geriatric assessment, nursing tools, older people care

Word count: Abstract (336); main text (4028), references (1580)

Contributing authors: Please insert your contact details

Authors	E-mail addresses	Address
Mr Massirfufulay Kpehe Musa	m.musa2@herts.ac.uk	Centre for Research in Public health and Community Care (CRIPACC) University of Hertfordshire AL10 9AB
Dr Gizdem Akdur	g.akdur@herts.ac.uk	Centre for Research in Public health and Community Care (CRIPACC) University of Hertfordshire AL10 9AB
Professor Barbara Hanratty	barbara.hanratty@newcastle.ac.uk	Population Health Sciences Institute Biomedical Research Building (Second floor) Campus for Ageing and Vitality Newcastle University Newcastle upon Tyne NE4 5PL
Dr Sarah Kelly	sarah.kelly@thisinstitute.cam.ac.uk	Institute of Public Health University of Cambridge CB2 0SR
Dr Adam Gordon	adam.gordon@nottingham.ac.uk	School of Medicine University of Nottingham DE22 3DT
Dr Guy Peryer	g.peryer@uea.ac.uk	Faculty of Medicine and Health Sciences,

For peer review only

For peer review only - http://bmjopen.bmj.com/site/about/guidelines.xhtml

		University of East Anglia, NR4 7TJ
Professor Karen Spilsbury	k.spilsbury@leeds.ac.uk	School of Healthcare, Faculty of Medicine and Health, University of Leeds, Leeds, LS2 9JT
Dr Anne Killett	a.killett@uea.ac.uk	Faculty of Medicine and Health Sciences<br>University of East Anglia<br>Norwich Research Park<br>NR4 7TJ
Dr Jennifer Burton	Jenni.Burton@glasgow.ac.uk	Institute of Cardiovascular and Medical Sciences, University of Glasgow, New Lister Building, Glasgow Royal Infirmary, G31 2ER
Professor Julienne Meyer	J.Meyer@city.ac.uk	National Care Forum
Ms Sue Fortescue	sfortescue@hotmail.co.uk	Alzheimer Society Research Network
Ms Ann-Marie Towers	a.towers@kent.ac.uk	Centre for Health Services Studies<br>University of Kent<br>CT2 7NF
Ms Lisa Irvine	l.irvine@herts.ac.uk	Centre for Research in Public health and Community Care (CRIPACC) University of Hertfordshire AL10 9AB
Professor Claire Goodman	c.goodman@herts.ac.uk	Centre for Research in Public health and Community Care (CRIPACC) University of Hertfordshire AL10 9AB

For peer review only

ABSTRACT

Introduction: Care homes provide nursing and social care for older people who can no longer live independently at home. In the UK, there is no consistent approach to how information about residents' medical history, care needs, and preferences are collected and shared. This limits opportunities to understand the care home population, have a systematic approach to assessment and documentation of care, identify care home residents at risk of deterioration, and review care. Countries with standardised approaches to residents' assessment, care planning and review (e.g. Minimum Data Sets (MDS)) use the data to understand the care home population, guide resource allocation, monitor services delivery and for research. The aim of this realist review is to develop a theory-driven understanding of how care homes staff implement and use MDS to plan and deliver care of individual residents.

Methods and analysis: A realist review will be conducted in three research stages.

Stage one will scope the literature and develop candidate programme theories of what ensures effective uptake and sustained implementation of an MDS.

Stage two will test and refine these theories through further iterative searches of the evidence from the literature to establish how effective uptake of an MDS can be achieved.

Stage three will consult with relevant stakeholders to test or refine the programme theory (theories) of how an MDS works at the resident level of care for different stakeholders and in what circumstances. Data synthesis will use realist logic of analysis to align data from each eligible article with possible context-mechanism-outcome (CMO) configurations, or specific elements that answer the research questions.

Ethics and dissemination: The University of Hertfordshire Ethics Committee has approved this study (HSK/SF/UH/04169). Findings will be disseminated through briefings with stakeholders, conference presentations, a national consultation on the use of an MDS in UK long-term care settings, publications in peer-reviewed journals, and in print and social media publications accessible to residents, relatives, and care home staff.

Review registration number: This review protocol is registered on the International Prospective Register of Systematic Reviews (PROSPERO) - CRD42020171323.

Keywords: Care Home, minimum data set, geriatric assessment, nursing tools, older people care

For peer review only

For peer review only - http://bmjopen.bmj.com/site/about/guidelines.xhtml

Strengths and limitations of this study

- The review will identify what needs to be in place to support the implementation of an MDS in long term care settings where standardised approaches to resident assessment and data collection are not in place.
- The review will demonstrate how using an MDS affects the everyday work and care practices of staff and its impact on residents' care.
- The synthesis will integrate qualitative and quantitative evidence that offers transferable learning for long term care settings that do not currently use an MDS.
- There are time constraints that may result in the team focusing on or prioritising some aspects of an MDS implementation over others.

BACKGROUND

There are nearly 12 million (11,989,322) people aged 65 years and above in the United Kingdom (UK) of which an estimated 1.6 million are aged 85 years and above, and more than 500,000 (579,776) people are aged 90 years and above.[1] However, with greater longevity (e.g. age 85 years and above) comes higher levels of dependency, dementia and comorbidity,[2] which in turn intensify the need for social care services.[3] Approximately 420,000 older people in England and Wales live in care homes. [4] Care home is a generic term that refers to facilities where a number of older people live together and have staff available 24 hours to provide personal care (e.g. residential care or assisted living/supportive housing facilities), and those facilities where a qualified nurse is required on duty 24 hours to provide additional nursing care for more dependent residents (e.g. nursing homes or skilled nursing facilities).[5] The care home population represents the oldest and most vulnerable group of older people,[6] with approximately 70% of them living with cognitive impairment, [6 7] and 76% requiring assistance with mobility.[8]

In the UK, there is no consistent approach to how information about residents' medical history, care needs, and preferences is collected and used. The absence of a national mandate, lack of links with National Health Services (NHS) data, and implementation challenges have meant that a minimum data set (MDS) and data-driven approaches to resident assessment have been limited to single projects.[9] The lack of a link between care home data and the NHS data recently became evident when figures reported by the Office for National Statistics during the first three weeks of Covid-19 underestimated the impact of the pandemic among care home residents.[10] All care homes, however, routinely collect

For peer review only

For peer review only - http://bmjopen.bmj.com/site/about/guidelines.xhtml

large amounts of data about their residents. The challenge is to establish systems of assessment and recording that are evidence-based, accessible and valuable to those using, providing, commissioning and regulating care services. Without a unified record there may be duplication of assessments, communication failures and, unmet care needs.[11 12] Over the next two decades the number of older people likely to need long term care will increase;[2] so determining consistent ways to assess and document care for residents in these settings is a priority.

A minimum data set (MDS) in this realist review is defined as a comprehensive, standardised account of the characteristics and needs and ongoing care of residents living in long-term care (care home) settings. The review concentrates on how an MDS is used by care home staff and what supports effective uptake for the benefit of individual residents. It also takes account of the involvement of residents themselves and their family in influencing how an MDS is used.

There are multiple versions of MDS, which are often country specific. However, all versions of MDS share a common language and are designed to support an integrated system of care that can support cross-sector clinical and managerial decision-making. One example of an MDS is the International Resident Assessment Instrument (InterRAI), developed for long term care facilities (LTCF).[13] The use of an MDS is often mandated and/or linked to national reimbursement systems and quality monitoring. Research has demonstrated the value of an MDS to commissioners and service providers in enabling identification of care needs and residents at risk of ill health.[14-18] They can provide a comprehensive account of resident characteristics, resource use, and care outcomes in key areas (e.g. activities of daily living, cognitive performance, pain, cost of care, and infection).[19] However, Kontos and colleagues argue that a standardised process such as the MDS fails to consistently result in individualised care planning, which may suggest problems with content of an MDS.[20]

For long term care settings making the transition to standardised approaches to data collection, little is known about what needs to be in place to implement an MDS and how its use impacts on staff work, time away from care, knowledge of the care home residents, working with other health care professionals and benefits (or not) to residents, staff and residents' families.

For peer review only - http://bmjopen.bmj.com/site/about/guidelines.xhtml

METHODS AND ANALYSIS

Review aim and objectives

Aim

To develop a theory-driven understanding of how care homes' staff can effectively implement and use MDS to plan and deliver care of individual residents.

Objectives

1. Develop a programme theory describing contexts that can support the uptake and use of an MDS in care homes.
2. Identify in what circumstances the use of an MDS produces improved outcomes (including resource use) for an individual resident, their family, and the care home staff and their employing organisation.

Study design

We will conduct a realist review which seeks to formulate, test and refine the programme theory while assessing whether and how the programme succeeds in the local setting,[21] in order to generate important insights for the United Kingdom. A programme theory is an overarching theory or model of how a programme, or an intervention is expected to work[22] and it helps to explain (some of) 'how and why, in the "real world", a specific programme "works", for whom, to what extent and in which contexts'.[23] The unit of analysis in a realist review is the ideas and assumptions (i.e., the programme theories) that underlie an intervention and explain how it works to achieve the desired outcomes.

A realist review is an interpretive, theory-driven approach to evidence synthesis[24 25] to develop a programme theory of the causal processes and context-specific factors that can explain how an intervention or programme is expected to work. Realism is a methodological paradigm which sits between positivism (the world is real and can be observed directly) and constructivism (given that all we know has been processed through the human mind, we can never be sure exactly what reality is).[26] It is flexible to changes and embedded in a social reality that influences how a programme is implemented and how various actors in that reality respond to it.[21]

Programmes like the minimum data set (MDS) will always rely on human agency to affect change. A realist approach argues that the features or elements of the programme will produce a range of

For peer review only - http://bmjopen.bmj.com/site/about/guidelines.xhtml

potential responses to the programme which will impact on the outcomes.[21 24] It assumes that there is a knowable, independent reality that will shape how different participants react to a programme, whether they are aware of these influences or not.[27] Thus, uptake and implementation of an MDS can lead to different outcomes for different stakeholders (e.g. residents and their relatives, staff, commissioners, regulators) depending on who is involved, the resources available and how the MDS is used.[28 29]

Using a realist approach,[21] there are four key linked concepts for explaining and building a theory of how a programme works: (i) contexts (C), which are often the 'backdrop' of interventions;[24] (ii) mechanisms (M), which are not observed directly but account for what it is about programmes that make them work,[30 31] characterised as *"a process that bring about or prevents some change in a concrete system"*[32] (iii) outcomes (O) of the intervention (planned or unplanned, visible or not) or strategies of the intervention;[33] It is the Context-Mechanism-Outcome (CMO) configurations (models indicating how programmes activate mechanisms for whom and in what conditions, to elicit outcomes) that are the building blocks of the theory.[25] Thus, in care home settings, staff understanding of their responsibility for completing an MDS could be a context (C), which triggers how staff prioritise recording information as part of care work (M) to identify residents at risk of deterioration (O).

The review will follow Pawson's five practical stages of conducting realist reviews: clarify the scope of the review, search for evidence, appraise primary studies and extract data, synthesise the evidence and draw conclusions, and disseminate the findings.[21] Organised in three stages, we will first undertake a scoping of the literature to identify care home specific work on the uptake of MDS and develop relevant theories around staff uptake and implementation and outcomes specific to the use of an MDS in long term (care home) settings. Stage two will test and refine the emergent theories that underpin the use of an MDS and that leads to both intended and unintended outcomes for staff and residents. Stage three will synthesise the findings to establish how and when the use of an MDS achieves different outcomes for residents, families, staff and organisations.

For peer review only - http://bmjopen.bmj.com/site/about/guidelines.xhtml

Stages of the review process

Stage 1: Defining the scope of the review, identifying existing theories and theory development

This review is nested within a larger review ("*A systematic review of process and contextual factors that influence research implementation in care homes and identification of key measures and outcomes in care home research*"; PROSPERO reference: CRD42020155923). The literature identified from the larger review will be the starting point for the scoping work.

The scoping of the literature will focus on studies that report on how an MDS is used in long term care (care home) settings. Outcomes of interest will be established by the project team as an iterative process but are likely to include evidence of its impact on: accuracy of reporting, needs assessment, staff workload, quality of care, resource use, staff satisfaction and access to care.

Literature search strategy

Searches for relevant evidence will include databases of peer-reviewed literature (MEDLINE, EMBASE, CINAHL, ASSIA (Applied Social Sciences Citation Index and Abstracts) and sources of grey literature (including OpenGrey and websites of organisations relevant to care homes and care of older people). Studies for inclusion will be limited to English language. We will search data from January 2009 to March 2020. These initial searches will be complemented by:

1. Searching of both lateral and forward citations of included papers paying particular attention to seminal papers on the uptake and use of an MDS in long term care settings;

2. Contact with experts who have developed and/or use an MDS.

The search strategy will be iterative because predetermined linear search strategies are unlikely to generate search results that are adequate for purposes of conducting knowledge-building and theory-generating reviews.[34] Throughout the proposed review and based on the scoping review findings we will introduce new , targeted search terms not defined in the initial searches.[34 35] We will use search terms such as care homes, nursing homes, and nursing homes (Supplementary Table S1). We will then combine these terms with other terms such as MDS, Minimum Data Set, Inter-RAI, Research Assessment Instrument, and RAI using Boolean logic (Supplementary Table S1). A comprehensive list

For peer review only - http://bmjopen.bmj.com/site/about/guidelines.xhtml

of search terms and databases used will be provided in subsequent publications on completion of the proposed research.

Literature screening process

Search results relevant to MDS will be downloaded into Covidence software. Screening and selection of articles will take place in two stages (title and abstract, and full text).[36] Two reviewers (MKM and GA) will independently screen titles and abstracts identified by electronic search and applied the selection criteria to potentially relevant full-text papers.[37] The two reviewers (MKM and GA) will then independently screen 10 articles and cross-check results to discuss emergent ideas and themes and establish consensus on the relevance of the documents. Disagreement between MKM and GA will be resolved by the third reviewer, CG.

Based on earlier work that used an MDS to collect data and cross team discussions,[38] the initial programme theory will focus on how an MDS is used in long term care. This will be the basis for scoping the literature on the challenges of changing systems of care, the need for a policy or regulator mandate, how it affects patterns of working in the care home, staff involvement in data entry and changes in residents' care. At this stage we will not be assuming causality but we will recognise that these are likely to influence uptake and use and resident and staff outcomes. Studies that have used MDS, or similar approaches to document resident, staff and organisational outcomes but do not address questions of implementation and use will be reviewed to identify supplementary evidence on related issues of interest (e.g. accuracy of data, time commitment and how information was used and by who). The search strategy will include citation searching and grey literature, and will be iteratively extended and refocused as the review progresses.

Formulating initial programme theories

At this stage, we will investigate demi-regularities in outcome patterns by developing a series of 'if-then statements' from the scoping literature to summarise the dominant arguments and supporting evidence of what supports the uptake and use of MDS in long-term care settings. This will inform the development of hypotheses that posit possible contexts (C) that are the backdrop to successful (or not) uptake[24] of MDS; the mechanisms (M) they trigger[32] and planned or unplanned Outcomes (O) arising from the use of MDS.[33] Possible context-mechanism-outcome (CMO) configurations will be

For peer review only - http://bmjopen.bmj.com/site/about/guidelines.xhtml

discussed across the research team and with subject experts on MDS and will inform stage two of the review phase and additional searches.

There are several ways to conceptualise the development and uptake of MDS as many have their roots in medical approaches to assessment and health systems design. It is therefore likely that the review will be informed by and aim to build on theories of implementation in long-term care,[32 39] uptake of technological innovation[40 41] assessment of older people with complex needs,[2 4 8] person-centred care,[42 43] and risk management and quality assurance.[44]

The introduction of an MDS in care homes is sensitive to the resource and policy constraints under which the care homes operate. Candidate theories will therefore consider the role of the regulator and legal frameworks that incentivise (or not) data sharing across organisations.

Literature selection, quality appraisal and data extraction criteria

There will be no restriction on the types of study design for eligibility.[45] Article selection will be based on the extent to which research on the uptake and routine use of an MDS can contribute to the development of a programme theory of implementation of MDS in long term care settings.

Included studies are likely to cover the following:

- Studies on the introduction and development of an MDS with care home staff;
- Studies that focus on the inclusion and engagement of care home staff, residents and their representatives in sharing resident data with the specific remit of improving resident outcomes;
- Implementation studies that provide evidence on what facilitates and inhibits the shared documentation and care planning in care home setting including digital innovation;
- Studies on commissioning services for care homes based on care home generated data;

For peer review only - http://bmjopen.bmj.com/site/about/guidelines.xhtml

No geographical restrictions will apply, although we will only include studies that are published in English and focus on the uptake and use of an MDS in long term care settings.

Quality appraisal of included articles

The quality of included papers will be carried out in accordance with previous appraisal work within a realist project.[29 36] The quality appraisal of included studies will be combined with data extraction, a technique usually employed in realist review.[46] Realist reviews employ various techniques to assess the quality of evidence by drawing on evidence from a wider range of sources unlike traditional systematic reviews that only focus on the methodological quality of studies.[29 36] As quality of evidence is not limited to the methodological quality, or hierarchy of evidence in realist reviews,[29] each article in this review will be assessed based on its trustworthiness and applicability to the research questions. Consistent with the realist approach, we will use an iterative approach to determine whether an article is considered "*good enough and relevant*" to answer our research questions.[47] Good enough will be based on the reviewers' own assessment of the quality of evidence, for example if it is considered to be of a sufficient standard for the research question, and relevance will relate to whether the authors provided sufficient descriptive detail and/or theoretical discussion to contribute to the initial programme theories development.[37]

The quality appraisal in this review will be assessed on a case by case basis considering the opportunities for learning, scientific rigor of evidence and relevance to the review questions.[46] Two reviewers (MKM and GA), in consultation with CG will lead this process. Weaker papers and those with equivocal or negative findings will be considered if they contribute to the overall programme theories.

Data extraction

Data extraction will be conducted on the basis of relevance to the review questions and will be based on realist guidelines to address questions that explore "what is it that supports (or hinders) an MDS implementation in care homes, and how care home staff use and interpret an MDS to guide residents' care?"[36] From the extracted data, two reviewers (MM and GA) will independently rate the studies as either yes, no, or maybe in terms of whether the particular article meets inclusion criteria. We will use

For peer review only - http://bmjopen.bmj.com/site/about/guidelines.xhtml

"maybe" for issues that cannot be answered based on the information available in the publication. Then the two reviewers (MM and GA) will meet with a third reviewer (CG) who will serve as an adjudicator to verify, confirm, or reject inclusion of the data. From relevant articles, several 'if-then' statements will be made from which initial programme theories will be made.

Data synthesis

Data synthesis of the scoping phase will use realist logic of analysis to align data from each eligible article with possible context-mechanism-outcome (CMO) configurations,[21] or specific elements that answer the research questions. These emerging findings, and putative patterns of association within the data will be tested further in stage two, to build causal explanations based on the observed interactions between context, mechanism, and outcomes.

Stage 2: Testing and refining the programme theories

Further iterative searches of the evidence will be directly informed by the CMOs developed in stage one as candidate programme theories. The iterative circle will continue throughout the course of the review until theoretical saturation has been achieved. [21 48]

Data will be extracted using a bespoke data extraction form. It will include descriptive data on study characteristics and is likely to focus on what can be learnt about the role and work of staff, the resources required to implement an MDS, the features of the settings (e.g., workforce capacity, size of care homes), explicit and implicit theories for how interventions were anticipated to work, and patient and carer outcomes. A sample of the papers, including those that appear to offer most learning and their completed data extraction forms will be shared across the project team to support ongoing discussion and debate of the candidate theory(ies) and their supporting evidence.

Stage 3: Analysis and synthesis of evidence from the proposed programme theories

To support hypothesis refinement and "fine tune" the theory(ies) that show the most promise, we will further test findings from stage two in a series of interviews.[49] We will do this through stakeholders' consultation (Box 1). It is acknowledged in realist research that published literature alone cannot help

For peer review only - http://bmjopen.bmj.com/site/about/guidelines.xhtml

to unearth the reasoning of end users of a programme.[21 26 31] Therefore, the inclusion of primary data from stakeholders in the review will be an added value.

We will carry out up to eight individual semi-structured interviews with frontline staff (staff from care homes who use predominately paper based records and staff who routinely use electronic records for their residents) and care home managers, and stakeholders who are experts regarding the use of care home residents' data. The semi-structured interviews will be guided by emerging programme theories from the early stages of the review.

Participants' selection will be purposive based on their knowledge of using MDS in and with care homes. All participants will be sent a detailed participant information sheet via email and consent form prior to the interview. Interviews will be either h face-to-face, online, or telephone conversations. Interviews will be audio recorded and transcribed.[21] Data will help to refine or refute the demi-regularities seen in outcome patterns emerging from the empirical literature.[26].

At the end of the interviews, we will present and discuss the programme theories, with the supporting evidence for discussion, with the whole research project team.

Box 1: Stakeholders' consultation

During stakeholders' consultation interviews, we will explore:

i) The fit between the emerging programme theories and how stakeholders understand what is needed for the development and use of MDS in long term care settings
ii) Alternative explanations stakeholders identify as relevant for the successful use of MDS by care home staff

A summary of the review process is presented in Figure 1. The double arrows within or between stages indicate iterative processes of the review.

Figure 1: The Realist Review Processes

For peer review only - http://bmjopen.bmj.com/site/about/guidelines.xhtml

The final programme theories will be synthesised narratively, by logic models, and/or summary tables where appropriate. The findings of the review will be written up according to the Realist And Meta-narrative Evidence Syntheses: Evolving Standards (RAMESES) guidance.[48]

Patient and Public Involvement

To keep the person being cared for at the centre of our thinking in ways that inform delivery of care or care home resident benefit, we will convene two care home based resident Patient and Public Involvement (PPI) groups that will meet throughout this review project. A member of this realist review, who is a former carer and IT specialist, will lead the PPI groups. We anticipate that input from residents and carers will help us to identify and understand the important contextual factors, and the resource and reasoning that support the implementation of an MDS in long term care settings. The PPI groups input will help us to tailor the stakeholders' consultation, inform our theory (or theories) development and ensure that the final refined programme theory(ies) resonates with care home staff and residents' experience.

DISCUSSION

This realist review will provide a theory-driven understanding of what needs to be in place for the successful implementation an MDS systems in care home settings to benefit residents, staff, families, service managers and commissioners.

Research has demonstrated the value of minimum data sets to commissioners and service providers in the identification of care needs.[50-54] A research study that used care home specific MDS identified specific implementation challenges.[38] It enabled comprehensive analysis of baseline resident data and residents at risk but there was limited staff capacity to support and sustain its completion over time when it was not linked to other data collection responsibilities.[38] This review addresses a gap in the evidence about what is needed to support uptake and implementation of an MDS, what needs to be in place for effective uptake and how an MDS is used in different circumstances to enable key care outcomes for residents. By identifying the causal mechanisms at work the review findings will directly inform decision-making about how to design, tailor and implement an MDS that is acceptable to staff and can inform residents' everyday care.

For peer review only - http://bmjopen.bmj.com/site/about/guidelines.xhtml

ETHICS AND DISSEMINATION

The University of Hertfordshire Ethics Committee has approved this study (HSK/SF/UH/04169). Findings will be disseminated through briefings with stakeholders, conference presentations, a national consultation on the use of an MDS in UK long-term care settings, and publications in peer-reviewed journals, and in print and social media publications accessible to residents, relatives, and care home staff.

FUNDING

This study/project is funded by the National Institute for Health Research (NIHR) Health Service Research and Delivery programme (HS&DR NIHR127234) and supported by the NIHR Applied Research Collaboration (ARC) East of England. This review started from December 2019 and it will end by 31st December 2020.

For peer review only

CONFLICT OF INTEREST

None

ACKNOWLEDGEMENT

Arne Wolters, Liz Jones and Iain Lang are collaborators on the Developing research resources And minimum data set for Care Homes' Adoption and use (DACHA) study and contributed to early conversations about the review and lead work in the larger study that will use these findings. We thank the reviewers Catherine Powell and Matthias Hoben whose constructive comments/suggestions helped to improve and clarify this manuscript.

DISCLAIMER

The views expressed are those of the author(s) and not necessarily those of the NIHR or the Department of Health and Social Care.

For peer review only - http://bmjopen.bmj.com/site/about/guidelines.xhtml

CONTRIBUTORS

Concept and design of the review are embedded in the Developing research resources And minimum data set for Care Homes' Adoption and use (DACHA) study. SK conducted the initial literature search and MKM, GA and CG wrote the first draft of the manuscript. Critical review and refinement of the manuscript was provided by GP, KS, AK, JB, BH, AG, JM, SF, AMT and LI. MKM, GA and CG approved the final version.

REFERENCES

1. Age UK. Later life in the United Kingdom 2019 2019 [Available from: https://www.ageuk.org.uk/globalassets/age-uk/documents/reports-and-publications/later_life_uk_factsheet.pdf accessed 08/05/2020.
2. Kingston A, Commas-Herrera A, Jagger C. Forecasting the care needs of the older population in England over the next 20 years: estimates from the Population Ageing and Care Simulation (PACSim) modelling study. *Lancet Public Health* 2018;3(9):e447-e55.
3. Kelly A, Kenny C. This POSTnote looks at the numbers of unpaid carers across the UK, and the amount and type of care provided. It examines the impacts of providing unpaid care on carers' education, employment, finances, health and wellbeing, and personal and social relationships. The note reviews the different types of support available for carers and examines evidence on their effectiveness. 2018 [Available from: https://post.parliament.uk/research-briefings/post-pn-0582/ accessed 08/05/2020.
4. Care Quality Commission (CQC). The state of health care and adult social care in England 2016/17: Care Quality Commission (CQC); 2017 [Available from: https://www.cqc.org.uk/sites/default/files/20171011_stateofcare1617_summary.pdf accessed 15/01/2020 2020.
5. Underwood M, Lamb SE, Eldridge S, et al. Exercise for depression in care home residents: a randomised controlled trial with cost-effectiveness analysis (OPERA). *Health technology assessment (Winchester, England)* 2013;17(18):1-281. doi: 10.3310/hta17180 [published Online First: 2013/05/02]

For peer review only - http://bmjopen.bmj.com/site/about/guidelines.xhtml

6. Musa M, Saga S, Blekken L, et al. The prevalence, incidence, and correlates of faecal incontinence among older people residing in care homes: a systematic review. *Journal of the American Medical Directors Association* 2019;20:956-62.
7. Alzheimer's Society. Facts for the media 2019 [Available from: https://www.ageuk.org.uk/globalassets/age-uk/documents/reports-and-publications/later_life_uk_factsheet.pdf accessed 15/01/2020 2020.
8. Gordon A, Franklin M, Bradshaw L, et al. Health status of UK care home residents: a cohort study. *Age and Aging* 2014;43(1):97-103.
9. Onder G, Carpenter I, Finne-Soveri H, et al. Assessment of nursing home residents in Europe: the Services and Health for Elderly in Long TERm care (SHELTER) study. *BMC Health Services Research* 2012;12(5):1-10.
10. Booth R. Hundreds of UK care home deaths not added to official coronavirus toll: The Guardian Newspaper 2020 [Available from: https://www.theguardian.com/world/2020/apr/09/covid-19-hundreds-of-uk-care-home-deaths-not-added-to-official-toll accessed 24/04/2020.
11. Age UK. General Election Manifesto 2019: Age UK; 2019 [Available from: https://www.ageuk.org.uk/globalassets/age-uk/documents/campaigns/ge-2019/age-uk-general-election-manifesto-2019.pdf accessed 15/01/2020 2020.
12. Iliffe S, Davies S, Gordon A, et al. Provision of NHS generalist and specialist services to care homes in England: review of surveys. *Primary Health Care Research & Development* 2015;17:122-37.
13. Hutchinson A, Milke D, Malsey S, et al. The resident assessment instrument-minimum data set 2.0 quality indicators: a systematic review. *Health Services Research* 2020;10:1-14.
14. Chen L, Liu L, Peng L, et al. Identifying residents at greater risk for cognitive decline by Minimum Data Set in long-term care settings. *Journal of Clinical Gerontology and Geriatrics* 2014;5(4):122-26.
15. Doupe M, Pass J, J N, et al. How well does the minimum data set measure healthcare use? a validation study. *BMC Health Services Research* 2018;18
16. Gleeny C, Stolee P. Comparing the functional independence measure and the interRAI/MDS for use in the functional assessment of older adults: a review of the literature. *BMC Getriatrics* 2009;9(52):1-12.
17. Hansebo G, Kihlgren M, Ljunggren G, et al. Staff views on the Resident Assessment Instrument, RAI/MDS, in nursing homes, and the use of the Cognitive Performance Scale, CPS, in different levels of care in Stockholm, Sweden. *Journal of Advanced Nursing* 1998;28(3):642-53.
18. Wysocki A, Thomas K, Mor V. Functional Improvement Among Short-Stay Nursing Home Residents in the MDS 3.0. *Journal of the American Medical Directors Association* 2015;16(6):470-74.
19. Carpenter G. Accuracy, validity and reliability in assessment and in evaluation of services for older people: the role of the interRAI MDS assessment system. *Age and Aging* 2006;35:327-29.
20. Kontos PC, Miller K-L, Mitchell GJ. Neglecting the importance of the decision making and care regimes of personal support workers: a critique of standardization of care planning through the RAI/MDS. *The Gerontologist* 2010;50(3):352-62. doi: 10.1093/geront/gnp165 [published Online First: 12/21]
21. Pawson R, Greenhalgh T, Harvey G, et al. Realist review - a new method of systematic review designed for complex policy interventiions *Journal of Health Services Research & Policy* 2005;10:21-34.
22. Maden M, Cunliffe A, McMahon N, et al. Use of programme theory to understand the differential effects of interventions across socio-economic groups in systematic reviews—a systematic methodology review. *Systematic Reviews* 2017;6(1):266. doi: 10.1186/s13643-017-0638-9

For peer review only - http://bmjopen.bmj.com/site/about/guidelines.xhtml

23. Wong G, Westhorp G, Manzano A, et al. RAMESES II reporting standards for realist evaluations. *BMC Medicine* 2016;14(1):96. doi: 10.1186/s12916-016-0643-1
24. Jagosh J, Pluye P, Wong G, et al. Critical reflections on realist review: insights from customizing the methodology to the needs of participatory research assessment. *Research Synthesis Methods* 2014;5(2):131-41.
25. Pawson R. Digging for nuggets: how 'bad' research can yield 'good' good evidence. *International Journal of Social Research Methodology* 2006;9(2):127-42.
26. Cooper C, Lhussier M, Shucksmith J, et al. Protocol for a realist review of complex interventions to prevent adolescents from engaging in multiple risk behaviours. *BMJ Open* 2017;7(9):e015477. doi: 10.1136/bmjopen-2016-015477
27. Clark A, Lissel S. Complex critical realism: tenents and application in nursing research. *Advances in Nursing Sciences* 2008;31(4):E67-E79.
28. Rahman A, Applebaum A. The nursing home minimum data set assessment instrument: manifest functions and unintended consequences - past, present, and future. *The Gerontologist* 2009;49(6):727-35.
29. Pawson R. Evidence-based policy: a realist perspective. London: Sage Publications Ltd 2006.
30. Gwyther H, Bobrowicz-Campos E, Luis Alves Apóstolo J, et al. A realist review to understand the efficacy and outcomes of interventions designed to minimise, reverse or prevent the progression of frailty. *Health Psychology Review* 2018;12(4):382-404. doi: 10.1080/17437199.2018.1488601
31. Rycroft-Malone J, Burton CR, Wilkinson J, et al. Collective action for implementation: a realist evaluation of organisational collaboration in healthcare. *Implementation Science* 2016;11(1):17. doi: 10.1186/s13012-016-0380-z
32. May C. Towards a general theory of implementation. *Implementation Science* 2013;8(18):1-14.
33. Jagosh J, Macaulay A, Pierre P, et al. Uncovering the Benefits of ParticipatoryResearch: Implications of a Realist Review forHealth Research and Practice. *The Milbank Quarterly* 2012;90(2):311-46.
34. Finfgeld-Connett D, Johnson ED. Literature search strategies for conducting knowledge-building and theory-generating qualitative systematic reviews. *J Adv Nurs* 2013;69(1):194-204. doi: 10.1111/j.1365-2648.2012.06037.x [published Online First: 2012/05/18]
35. Papaioannou D, Sutton A, Carroll C, et al. Literature searching for social science systematic reviews: consideration of a range of search techniques. *Health information and libraries journal* 2010;27(2):114-22. doi: 10.1111/j.1471-1842.2009.00863.x [published Online First: 2010/06/23]
36. Abayneh S, Lempp H, Manthorpe J, et al. Development of programme theory for integration of service user and caregiver involvement in mental health system strengthening: protocol for realist systematic review. *Int J Ment Health Syst* 2018;12:41-41. doi: 10.1186/s13033-018-0220-4
37. Bunn F, Goodman C, Russell B, et al. Supporting shared decision making for older people with multiple health and social care needs: a realist synthesis. *BMC Geriatrics* 2018;18(1):165. doi: 10.1186/s12877-018-0853-9
38. Goodman C, Davies S, Gordon A, et al. Optimal NHS service delivery to care homes: a realist evaluation of the features and mechanisms that support effective working for the continuing care of older people in residential settings. *Health Services and Delivery Research* 2017;5(29) doi: 10.3310/hsdr05290
39. Nilsen P. Making sense of implementation theories, models and frameworks. *Implementation Science* 2015;10(53) doi: 10.1186/s13012-015-0242-0
40. Department of Health & Social Care. Policy paper: The future of healthcare: our vision for digital, data and technology in health and care: Department of Health & Social Care; 2018 [Available from: https://www.gov.uk/government/publications/the-future-of-healthcare-our-vision-for-digital-data-and-technology-in-health-and-care accessed 26/03/2020.

For peer review only - http://bmjopen.bmj.com/site/about/guidelines.xhtml

41. Hall A, Brown Wilson C, Stanmore E, et al. Implementing monitoring technologies in care homes for people with dementia: A qualitative exploration using Normalization Process Theory. *International Journal of Nursing Studies* 2017;72:60-70.

42. McCormack B, McCance T. Person-centred Nursing: Theory and Practice. Oxford: Wiley Blackwell 2017.

43. Mitchell G. Person-Centred Care 2015 [Available from: https://blogs.bmj.com/ebn/2015/11/16/person-centred-care/ accessed 30/01/2020 2020.

44. Health Service Executive (HSE). Quality & Risk Management Standard 2007 [Available from: https://www.hse.ie/eng/about/who/oqr009-20080221-v3-quality-and-risk-management-standard.pdf accessed 26/03/2020.

45. Wong G, Greenhalgh T, Westhrop G, et al. Development of methodological guidance, publication standards and training materials for realist and meta-narrative reviews: the RAMESES (Realist And Meta-narrative Evidence Syntheses – Evolving Standards) project. *Health Services and Delivery Research* 2014;2(30)

46. Greenhalgh J, Pawson R, Wright J, et al. Functionality and feedback: a protocol for a realist synthesis of the collation, interpretation and utilisation of PROMs data to improve patient care. *BMJ Open* 2014;4(7):e005601. doi: 10.1136/bmjopen-2014-005601

47. Rycroft-Malone J, Burton C, Hall B, et al. Improving skills and care standards in the support workforce for older people: a realist review. *BMJ Open* 2014;4(5):e005356. doi: 10.1136/bmjopen-2014-005356

48. Wong G, Greenhalgh T, Westhrop G, et al. Development of methodological guidance, publication standards and training materials for realist and meta-narrative reviews: the RAMESES (Realist And Meta-narrative Evidence Syntheses – Evolving Standards) project. *Health Services and Delivery Research* 2014;2

49. Manzano A. The craft of interviewing in realist evaluation. *Evaluation* 2016;22(3):342-60. doi: 10.1177/1356389016638615

50. Carpenter I, Perry M, Challis D, et al. Identification of registered nursing care of residents in English nursing homes using the Minimum Data Set Resident Assessment Instrument (MDS/RAI) and Resource Utilisation Groups version III (RUG-III). *Age and Aging* 2003;32(3):279-85.

51. Hirdes J, Mitchell L, Maxwell C, et al. Beyond the 'iron lungs of gerontology': using evidence to shape the future of nursing homes in Canada. *Canadian Journal on Aging/la Revue canadienne du vieillissement* 2011;30(3):371-90.

52. Jamieson H, Nishtala P, Scrase R, et al. Drug burden and its association with falls among older adults in New Zealand: a national population cross-sectional study. *Drug & Aging* 2018;35(1):73-81.

53. Ogarek J, McCreedy E, Thomas K, et al. Minimum Data Set Changes in Health, End-Stage Disease and Symptoms and Signs Scale: A Revised Measure to Predict Mortality in Nursing Home Residents. *Journal of American Getriatric Society* 2018;66(5):976-81.

54. Szczerbińska K, Hirdes J, Zyczkowska J. Good news and bad news: depressive symptoms decline and undertreatment increases with age in home care and institutional settings. *The American Journal of Geriatric Psychiatry* 2012;20(12):1045-56.

For peer review only - http://bmjopen.bmj.com/site/about/guidelines.xhtml

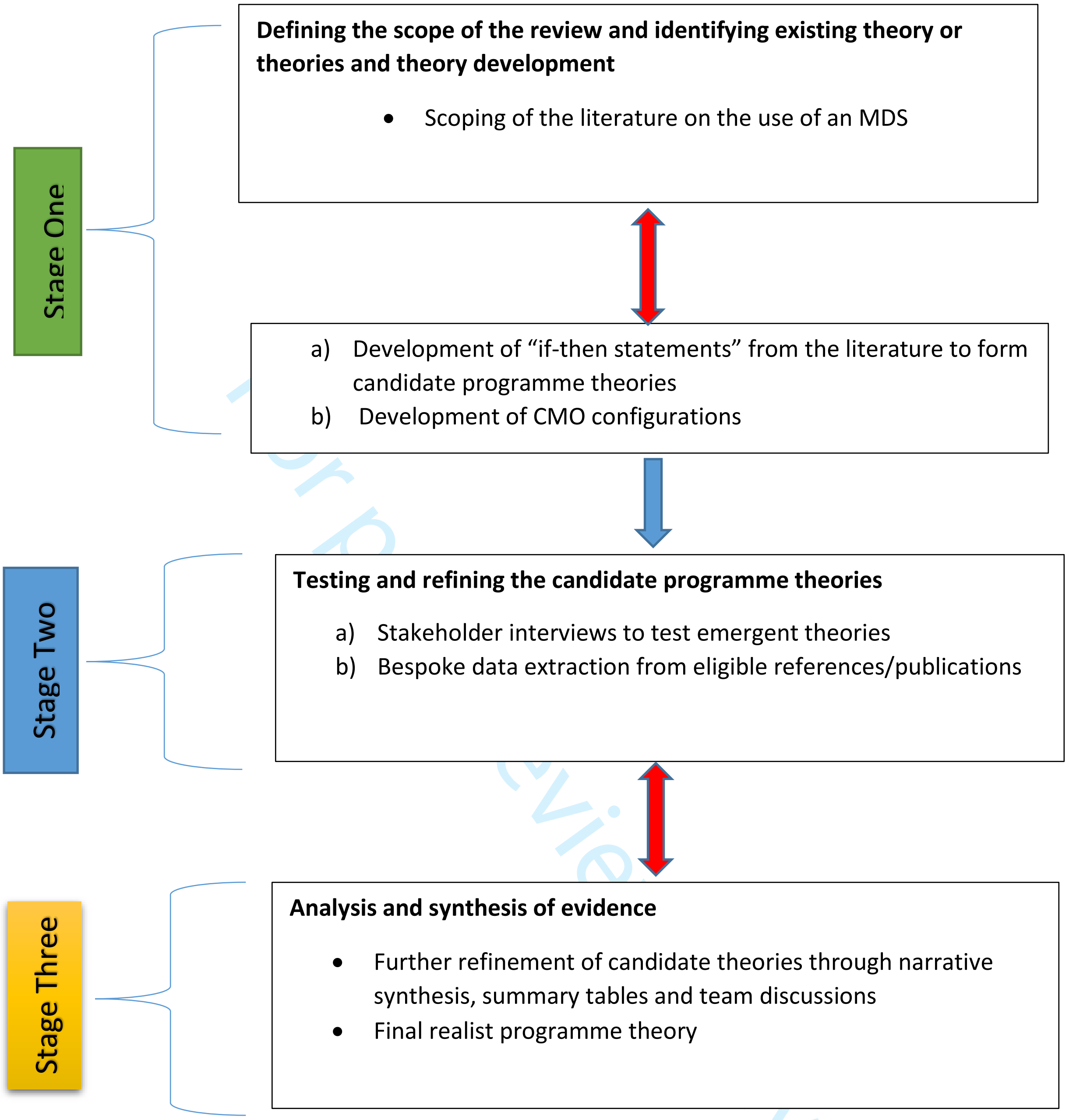
Stage One
Defining the scope of the review and identifying existing theory or theories and theory development
• Scoping of the literature on the use of an MDS
a) Development of "if-then statements" from the literature to form candidate programme theories
b) Development of CMO configurations
Stage Two
Testing and refining the candidate programme theories
a) Stakeholder interviews to test emergent theories
b) Bespoke data extraction from eligible references/publications
Stage Three
Analysis and synthesis of evidence
• Further refinement of candidate theories through narrative synthesis, summary tables and team discussions
• Final realist programme theory

Supplementary Table S1: Proposed search terms for care homes minimum data set (MDS)

1. Combined searches for scoping review and process evaluation review (DACHA Study's workpackage-1)

**1. *Homes for the Aged/**
**2. *Nursing Homes/**
**3. *Long-Term Care/**
**4. *Residential Facilities/**
**5. *Respite Care/**
**6. *Intermediate Care/**
**7. "care home$".ab,ti.**
**8. "nursing home$".ab,ti.**
**9. "residential care".ab,ti.**
**10. ("long term care" or "long-term care" or "longterm care").ab,ti.**
**11. "home$ for the aged".ab,ti.**
**12. "care facilit*".ab,ti.**
**13. "old$ people$ home$".ti,ab.**
**14. (retir$ adj2 home$).ab,ti.**
**15. ("old$ adult$" adj3 (facilit$ or residential or accommodation)).ab,ti.**
**16. ("old$ people$" adj3 (facilit$ or residential or accommodation)).ab,ti.**
**17. ("old$ person$" adj3 (facilit$ or residential or accommodation)).ab,ti.**
**18. ((geriatric$ or elder$ or senior$ or retir$) adj3 (facilit$ or residential or accommodation)).ab,ti.**
**19. "respite care".ti,ab.**
**20. "intermediate care".ti,ab.**
**21. or/1-20**
**22. *Randomized Controlled Trials as Topic/**
**23. Randomized controlled trial/**
**24. Random allocation/**
**25. Double blind method/**
**26. Single blind method/**
**27. Clinical Trial/**
**28. Clinical trials as Topic/**
**29. "randomi*ed".ab,ti.**
**30. randomly.ab,ti.**
**31. controlled clinical trial.pt.**
**32. Evaluation Study/**
**33. Comparative Study/**
**34. "before and after study".ti,ab,mp.**
**35. or/22-34**
**36. "Outcome and Process Assessment (Health Care)"/**
**37. *Implementation Science/**
**38. "process evaluation".ab,ti.**
**39. (process$ adj3 evaluation$).ab,ti.**
**40. (program$ adj3 evaluation$).ab,ti.**
**41. implementation.ab,ti.**
**42. context$.ab,ti.**
**43. fidelity.ab,ti.**
**44. or/36-43**
**45. *Qualitative Research/**
**46. *Focus Groups/**
**47. *Interviews as Topic/**
**48. *Narration/**
**49. (("semi-structured" or semistructured or unstructured or informal or "in-depth" or "indepth" or "face to face" or structured or guide) adj3 (interview$ or discussion$ or questionnaire$)).ab,ti.**
**50. or/45-49**
**51. 44 or 50**
**52. 21 and 35**
**53. 51 and 52**
**54. limit 52 to yr="2009 -Current"**
**55. 53 or 54**

For peer review only

2. Hits from the above searches will be downloaded into Endnote; we will then search in Endnote for MDS related studies using the following terms:

MDS
Minimum Data Set
Minimum Data
Inter-RAI
RAI
Research Assessment Instrument

For peer review only
